# Improving care at scale: process evaluation of a multi-component quality improvement intervention to reduce mortality after emergency abdominal surgery (EPOCH trial)

**DOI:** 10.1186/s13012-018-0823-9

**Published:** 2018-11-13

**Authors:** T. J. Stephens, C. J. Peden, R. M. Pearse, S. E. Shaw, T. E. F. Abbott, E. Jones, D. Kocman, G. Martin, on behalf of the EPOCH trial group, Simon Fletcher, Pieter Bothma, Ayodele Obideyi, Vivek Chitre, Dhiraj Ali, Richard Howard-Griffin, Vlad Kushakovsky, Michael Crabtree, Stephanie Bell, Vishal Patil, Asif Jah, Razeen Mahroof, Mark Blunt, Alistair Steel, Surjait Singh, Helen Porter, Helen Agostini, Matthew Tutton, Ayres Caldeira, Debbie Campbell, Dilshan Arawwawala, Thomas Pearson, Ben Maddison, Katherine Rowe, Chris Morris, Tanuja Shah, Gillian Tierney, John Williams, Lynsey Judd, Krishnamurthy Badrinath, Nicholas Watson, Gareth Moncaster, Sonia Gill, John Tansley, Victoria Banks, Jonathan Mole, John Abercrombie, Amit Shukla, Catherine ODwyer, Adam Wolverson, Tanweer Ahmed, Sarah Ford, Elizabeth Clements, Maria Tute, Tim White, Sarah Beavis, Sue Glenn, Neil Flint, Marcus Wood, Andrew Miller, Dawn Hales, Paul Hayden, Nandita Divekar, Neil Kukreja, Kirti Mukherjee, Somi Desikan, Tim Campbell-Smith, Simon Parrington, Vesna Hogan, Christie Locke, Anne Shears, Greg Lawton, Lee Baldwin, Simon Bailey, Kenneth Adegoke, Nat Natarajan, Mansoor Akhtar, Mansoor Sange, Mallikarjunappa Satisha, Mark Watson, Matthew Gardner, B. Aravind, Daniel Conway, Kevin Sim, Amanda Mccairn, Michael Chadwick, Preeti Kuduvalli, Jane Parker, Michael Raraty, Chris Brearton, Lawrence Wilson, Nicole Robin, Anita Jhamatt, Dale Vimalachandran, Peter Alexander, Jon Hopper, Abhiram Sharma, Oliver Hill, Andrew Brennan, Stephen Fletcher, John Griffith, Sarah Buckley, Alastair Rose, Sandeep Varma, Christopher Macklin, Michael Machesney, Ashok Raj, Abdul Nazar, Hitesh Patel, Otto Mohr, Dolores Mateo, Nicholas Bunker, Davina Ross-Anderson, Charles Knowles, Ajit Abraham, Tomas Jovaisa, Oluremi Odejinmi, Dipankar Mukherjee, Susan Jain, Tabitha Tanqueray, Tamzin Cuming, Ramani Moonesinghe, Michael Patterson, Jonathan Mccullough, Sanjiv Patel, Amir Rafi, James Limb, Andrew Mitchell, Alistair Roy, Robert Corson, Sean Cope, Elizabeth Hall, Bruce Gibson, James Brown, Sara Pick, Matthew Gaughan, Yvonne Marriott, Mark Eltringham, Vanessa Linnett, Anita Holtham, Sophie Noblett, Chris Dawson, Andrew Mitchell, David Saunders, Ian Clement, Stefan Plusa, Diane Monkhouse, Jost Mullenheim, Peter Davis, Emanuel Cirstea, Mike Bradburn Fiona McMenemie, Anton Krige, Daren Subar, Dominic Sebastian, Robert Shawcross, Emma Brennan, Cupitt, Thompson, Harper, Helen Spickett, Jonathan Barker, Emma Davies, Chris Coldwell, Mark Wilkinson, Heather Pratt, Panna Patel, Jyrki Karvonen, Gillian O’connell, Sean McAfee, Wael Khalaf, Christopher Lewis, Thomas Owen, Keiarash Jovestani, Arnab Bhowmick, Emma Durant, Sean Mcmullan, Banwari Agarwal, Rovan Dsouza, Daniel Martin, Omar Faiz, Tamsin Rope, Tariq Husain, J. Warusavitarne, Paul Ziprin, Martin Stotz, Glenn Arnold, Rachel Bartlett, Ruth Griffin, Andrew Thorniley, Alistair Myers, Nicola Stranix, Francesca Rubulotta, Tim Geary, Colin Pow, Gary Nicholson, Dewi Williams, David Wayne Wrathall, Alan Morrison, Gavin Bryce, Khaled Razouk, Kathryn Cain, Michael Gillies, Kevin Rooney, Jennifer Edwards, Susan Moug, Malcolm Sim, Paul Harrison, Christopher Wilson, Steven Henderson, Gudrun Kunst, Phil Hopkins, Stavros Papagrigoriadis, David Melville, Maurizio Cecconi, Peter Holt, Richard Hartopp, Adrian Fawcett, Amira Girgis, Britta O’Carroll-Kuehn, Stella Vig, Justin Woods, Isabella Karat, Stanislaw Jankowski, Samiy Farhat, Alastair Ankers, Rame Sunthares, Matthew Outram, Wilkinson Jonny, Guy Finch, Deborah Shaw, Marion Jonas-Obichere, Giovanni Brescia, Stapleton Clare, Roy Fernandes, Stephen Baxter, Malcolm Watters, Julian Stone, Christopher Thorn, Andrew White, Nikolaos Makris, Anil Hemandas, Tim HAVARD, Valerie Hilton, Huw Davis, Majd Aalshamaa, Piroska Toth-Tarsoly, Alexandra Scott, Xavier Escofet, Babu Muthuswamy, Gethin Williams, Michael Martin, Ajit Sivasankaran, Mark Henwood, Gordon Milne, Edward Curtis, Tom MorganJones, Krishnamurthy Somasekar, Richard Pugh, Ramesh Rajagopal, Shrisha Shenoy, Lucie Hobson, Stuart Mercer, Aneeta Sinha, Vanessa Tucker, James Kirkby-bott, Jenny McLachlan, Carolyn Way, Mark Edwards, Lynsey Houlton, Simon Sleight Belinda Cornforth, Louise Bell, Philip Dodd, Fenella Welsh, Geoff Watson, Gary Minto, Sam Waddy, Iain Christie, Richard Gibbs, Tom Edwards, Hamish Noble, Guy Rousseau, Jan Hanousek, Mark Cartmell, Rachael Craven, Jane Blazeby, Dan Freshwater-turner, Phoebe Syme, Mark Pulletz, Sarah Moreton, Anjay Talwar, Susie Baker, Jonathan Paddle, Alison Pickford, Denzil May, Robert Sutcliffe, Taj Saran, Roger Townsend, Gabriele Marangoni, Andrew Burtenshaw, Jaime Greenwood, Stephen Lake, Sam Sangal, Olga Tucker, Jeremy Marwick, Simon Smart, Jaysimha Susarla, Emma Leno, Kathryn Gill, Neil Cruickshank, Julian Sonksen, Raj Patel, David Stanley, Adrian Jennings, Andrew Claxton

**Affiliations:** 1 William Harvey Research Institute, Queen Mary University of London, London, UK; 2 Keck School of Medicine, University of Southern California, Los Angeles, USA; 3 Nuffield Department of Primary Care Health Sciences, University of Oxford, Oxford, UK; 4 Warwick Clinical Trials Unit, University of Warwick, Coventry, UK; 5 SAPPHIRE Group, Department of Health Sciences, University of Leicester, Leicester, UK; 6 THIS Institute (The Healthcare Improvement Studies Institute), University of Cambridge, Cambridge, UK; 7 Critical Care and Perioperative Medicine Research Group, Adult Critical Care Unit, Royal London Hospital, London, E1 1BB UK

**Keywords:** Emergency surgery, Quality improvement, Complex interventions, Evaluation

## Abstract

**Background:**

Improving the quality and safety of perioperative care is a global priority. The Enhanced Peri-Operative Care for High-risk patients (EPOCH) trial was a stepped-wedge cluster randomised trial of a quality improvement (QI) programme to improve 90-day survival for patients undergoing emergency abdominal surgery in 93 hospitals in the UK National Health Service.

**Methods:**

The aim of this process evaluation is to describe how the EPOCH intervention was planned, delivered and received, at both cluster and local hospital levels. The QI programme comprised of two interventions: a care pathway and a QI intervention to aid pathway implementation, focussed on stakeholder engagement, QI teamwork, data analysis and feedback and applying the model for improvement. Face-to-face training and online resources were provided to support senior clinicians in each hospital (QI leads) to lead improvement. For this evaluation, we collated programme activity data, administered an exit questionnaire to QI leads and collected ethnographic data in six hospitals. Qualitative data were analysed with thematic or comparative analysis; quantitative data were analysed using descriptive statistics.

**Results:**

The EPOCH trial did not demonstrate any improvement in survival or length of hospital stay. Whilst the QI programme was delivered as planned at the cluster level, self-assessed intervention fidelity at the hospital level was variable. Seventy-seven of 93 hospitals responded to the exit questionnaire (60 from a single QI lead response on behalf of the team); 33 respondents described following the QI intervention closely (35%) and there were only 11 of 37 care pathway processes that > 50% of respondents reported attempting to improve. Analysis of qualitative data suggests QI leads were often attempting to deliver the intervention in challenging contexts: the social aspects of change such as engaging colleagues were identified as important but often difficult and clinicians frequently attempted to lead change with limited time or organisational resources.

**Conclusions:**

Significant organisational challenges faced by QI leads shaped their choice of pathway components to focus on and implementation approaches taken. Adaptation causing loss of intervention fidelity was therefore due to rational choices made by those implementing change within constrained contexts. Future large-scale QI programmes will need to focus on dedicating local time and resources to improvement as well as on training to develop QI capabilities.

**EPOCH trial registration:**

ISRCTN80682973 10.1186/ISRCTN80682973 Registered 27 February 2014 and Lancet protocol 13PRT/7655.

**Electronic supplementary material:**

The online version of this article (10.1186/s13012-018-0823-9) contains supplementary material, which is available to authorized users.

## Background

There is widespread recognition of the need to improve the quality and safety of peri-operative care globally [1, 2]. Data demonstrate both the volume of adverse events and complications related to surgery, and the need to focus on high-risk patients who suffer disproportionate morbidity and mortality [3–6]. Our group led a major trial to assess the clinical effectiveness of a quality improvement programme on outcomes for patients undergoing emergency abdominal surgery, also known as emergency laparotomy [7]. Emergency laparotomy is a commonly performed surgical procedure, with a high 30-day mortality [8–11] and wide variations in the standards of care delivered [10–12]. Previous small studies of quality improvement in this area have improved care delivery and reduced mortality without increasing costs [10, 13, 14]. The EPOCH trial was designed to establish whether a quality improvement approach could reduce mortality and length of hospital stay for this patient group, when delivered at a national level. The trial was performed against the backdrop of a national focus in the UK on emergency laparotomy and the launch of the National Emergency Laparotomy Audit (NELA) which began patient data collection 4 months before the start of trial recruitment [15].

**Fig. 1:**
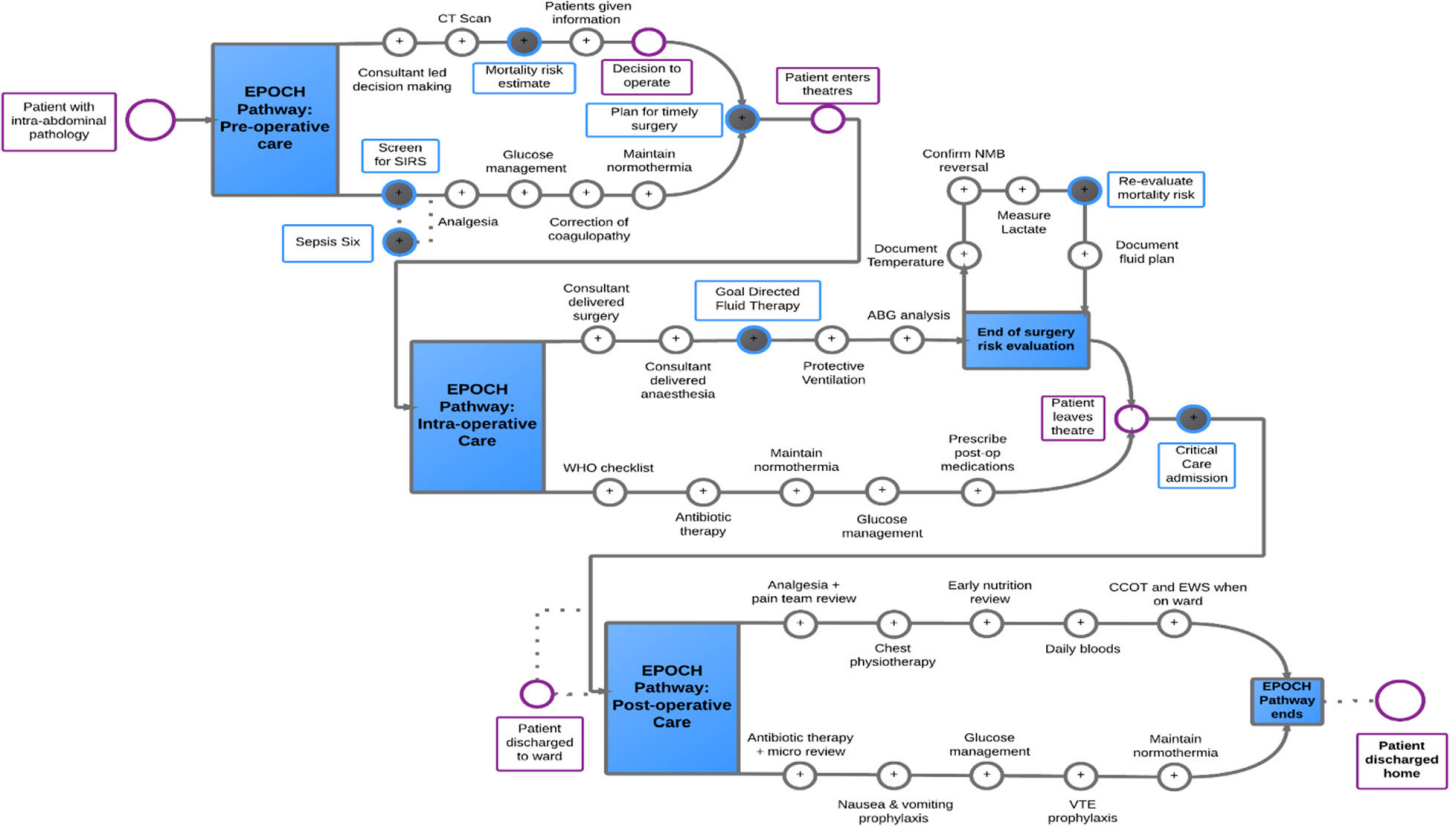
The EPOCH trial recommended care pathway. Legend: SIRS, systemic inflammatory response syndrome; Sepsis Six, a protocolised treatment for sepsis; CT, computer-aided tomography; WHO, World Health Organization; ABG, arterial blood gas; NMB, Neuro-muscular blockade; CCOT, critical care outreach team; NEWS, National Early Warning Score; VTE, venous thrombo-embolism

Quality improvement programmes can be seen has having a ‘hard core’, the clinical processes or practices that are the focus of improvement, and a ‘soft periphery’, the improvement methods that will enable change to occur [16]. In the EPOCH trial, the ‘hard core’ was a set of recommended clinical processes, organised within a care pathway for patients undergoing emergency laparotomy. The EPOCH trial care pathway was developed through an evidence-based Delphi consensus process to update existing guidelines published by the Royal College of Surgeons of England [17]. Details of the 37 component interventions are provided in Fig. 1, and a full summary of evidence grading is available on the trial website (www.epochtrial.org). The quality improvement intervention (the ‘soft periphery’) was designed to enable the QI leads and their teams to effectively improve the care pathway for patients undergoing emergency laparotomy. Two clinicians with quality improvement and training expertise (TS and CP) developed the programme theory (describing ‘the how’ and ‘the why’ of the QI intervention; see Tables 1 and 2) based on current evidence and learning from other quality improvement programmes [13, 18–20]. Quality improvement (QI) interventions, such as those delivered within the EPOCH trial, are complex due to their interacting components and the multiple organisational and social levels at which change must be effected [21, 22]. Delivering a complex intervention into a complex system, such as the perioperative care pathway in a hospital, is challenging with many possible barriers to achieving intended outcomes. Even within a trial setting, this complexity may mean that the target group is not actually exposed to the planned interventions [21, 23]. Therefore, in addition to the main trial, we conducted a process evaluation, with the aim of providing greater understanding of the complexity inherent in large-scale improvement programmes such as the EPOCH trial.

**Table 1:** Summary of the EPOCH trial programme theory

If	
- Relevant data are reviewed and fed back to teams regularly,	
- Key professionals come together to form an improvement team,	
- QI leads and colleagues learn basic quality improvement approaches, and	
- Relevant stakeholders are made aware of the project and improvement goals	
Then	
- A shared view of performance and improvement gaps can be created,	
- Professionals can work as a team to define and achieve local improvement goals,	
- Basic quality improvement approaches can be employed to achieve the improvement goals, and	
- Stakeholders will be more engaged in the need for change and aware of how improvement will occur	
So that	
- Improvements in care delivery in line with the recommended care pathway can be achieved	
So that	
- Mortality after emergency laparotomy can be reduced.

**Table 2:** The EPOCH trial Quality Improvement (QI) programme theory

Desired outcomes	QI strategies	QuIP activities and resources	Evidence for inclusion within programme theory
Motivation for change created amongst stakeholders and improvement goals clearly understood	QI leads hold a stakeholder meeting after activation (QI strategy 1)	1. Pre-activation checklist (providing guidance for planning of stakeholder meeting) 2. Evidence for QI and need for change provided 3. Presentation on achieving engagement	• Improvement projects require attention to the social context in which improvements are to be made which in turn requires relevant stakeholders to be informed and engaged (e.g. evidence from both Michigan Keystone and Enhanced Recovery programmes [19, 50]) • Data feedback can create cognitive dissonance if it is at variance from self-assessed or perceived performance, which in turn can lead to motivation for change [51].
Inter-professional collaboration (IPC) fostered	Each hospital to form an inter-professional improvement team (QI strategy 2)	4. Team approach promoted 5. QI leads encouraged to invite colleague to EPOCH meetings 6. EPOCH VLE open to all local QI team members	• There is sound theoretical and empirical evidence for the specific role of clinically-led quality improvement teams in successful QI [44, 52].
Shared view of current performance created (‘situational awareness’)	QI leads analyse their own data (NELA data +/− case note reviews and local audit data) and feed this back to colleagues regularly (QI strategy 3)	7. Case-note review tool 8. Training on data for improvement 9. Training on how to access and analyse NELA data 10. Excel workbook programmed to create run charts from NELA data 11. Secure data sharing site created on VLE	• Creating situational awareness regarding clinical performance is seen as fundamental to The Model for Improvement [53] and is the foundation of Feedback Intervention Theory [51, 54] • Recent empirical data points to data feedback as central to success of several key QI projects [13, 19, 55] • Cochrane reviews on data feedback indicate a positive impact on quality improvement if feedback is appropriate and timely and when a path to improvement is proposed [37, 54].
Frontline teams develop and use basic QI skills to effect change	QI leads and other team members: Use time-series charts (‘run-charts’) (QI strategy 4) Segment the patient pathway (Qi strategy 5) Use the Plan-Do-Study-Act (PDSA) cycles (QI strategy 6)	12. Introduction to QI skills training provided 13. Links to further reading and training resources for QI 14. Telephone and email support	• Application of improvement science approaches such as the Model for Improvement require at least some basic skill acquisition, and evidence points to a deficit in this area putting significant strain on the ability of an improvement project to achieve its potential [40, 56]. • Time-series charts (‘run-charts’) are a simple and robust method of analysing and presenting (for data feedback) changes to care processes [57]. • Segmentation of the proposed patient pathway involves introducing interventions within the pathway in an iterative fashion. Pathway segmentation makes the clinical element of this intervention less complex, more compatible with current systems and may makes process changes more trial-able and lower risk [36] • The IHI’s Model for Improvement, incl. The PDSA cycle, is an internationally accepted approach to quality improvement [53, 58].

*QuIP* Quality Improvement Programme, *VLE* Virtual Learning Environment, *NELA* National Emergency Laparotomy Audit)

In this paper, we describe how one of the largest trials of a quality improvement intervention to date was planned, delivered and received across 93 hospitals that offer emergency abdominal surgery within the United Kingdom’s (UK) National Health Service (NHS), [15] and provide detailed analysis to facilitate a greater understanding of the main trial results.

## Methods

### Process evaluation

We undertook a mixed-methods process evaluation with both prospective and retrospective components, based upon recommended guidance for evaluation of cluster trials [21, 24]. All components of the evaluation were performed without knowledge of the trial results, either by participants or evaluators.

### Overview of the EPOCH trial

The EPOCH trial was a stepped-wedge cluster randomised trial across 93 UK National Health Service (NHS) hospitals. Patients were recruited from March 2014 to October 2015. Recruited hospitals were grouped into 15 clusters of six to eight geographically co-located hospitals. Data for the trial were obtained via the National Emergency Laparotomy Audit (NELA), funded separately by the UK Healthcare Quality Improvement Programme [25] which started collecting data on 1 December 2013. Each recruited hospital nominated three senior clinicians (consultants) to act as quality improvement leads (QI leads) from key clinical areas (surgery, anaesthesia and critical care) and confirmed NHS Trust executive board support. Improvement skills or previous improvement experience were not pre-requisites to be a QI lead. No QI leads received funded time to undertake the improvement work nor to attend study meetings. The EPOCH trial was approved by the Research Ethics Committee of the National Health Service (REC reference 13/EM/0415).

Six QI strategies were recommended to support pathway implementation: (1) stakeholder engagement, (2) building a QI team, (3) analysing local data collected for NELA, (4) using run-charts to inform progress and feedback to colleagues, (5) segmenting the patient pathway to make change more manageable, and (6) use of Plan-Do-Study-Act (PDSA) cycles to support the change process. The QI programme provided guidance on how to use the six QI strategies to implement the pathway; each cluster received an introductory day of QI training (at the cluster activation meeting), a follow-up half-day meeting and support from the trial quality improvement co-ordinator plus access to web-based resources designed for the programme. Nominated QI leads were encouraged to invite colleagues from their sites to join them at these meetings. To further create a collaborative environment for hospitals to share learning, two additional national meetings, we convened which teams in activated sites could attend [see Additional file 1 for full programme details]. The QI intervention was designed to be ‘light touch’, recognising the limited resources of the study, of clinician time within the NHS and the fact that data collection through NELA was already occurring. All sites received a small payment (£3700) to support local QI efforts, and ongoing QI advice was available by telephone or email from the programme leads (TS and CP).

### Data sources and data collection

Table 3 details the evaluation foci and the three data sources used to investigate these: (1) routine QI programme activity data (records of meeting attendance and use of the web-based resources), (2) data from an exit questionnaire sent to all QI leads and (3) ethnographic data. The 37-item, online questionnaire, administered at the end of the study period, was designed to allow QI leads to describe activities undertaken as well as their overall experience of leading the improvement projects. This provided information on fidelity to the intended intervention and what helped and hindered progress. The questionnaire included categorical yes/no answers and space for comments (see Additional file 1 for a full list of questions). The questionnaire was designed and piloted in line with best practice, with two rounds of testing using research team members, for readability and usability and a final round of testing using eight QI leads [26, 27]. Changes from this final round were very minor, and therefore, responses from this sample were included in the analysis. Only one response was required per hospital, but QI leads were asked to complete the questionnaire with colleagues. A pre-planned ethnographic evaluation was undertaken in six trial sites by researchers outside the main EPOCH team (GM, DK). A maximum variation sample of sites was chosen, with criteria focussed on size of the hospital, surgical volume and discipline of the QI lead. Periods of observation were scheduled, and interviews with clinicians were held at several points during the trial to monitor progress and reflect on what had been achieved and what had impeded progress. All interviews were audio recorded, and field notes recorded in a diary at the time of observation, or immediately afterwards. Further details of the ethnographic methods are reported elsewhere [28].

**Table 3:** Data collected for process evaluation

Aspect of process evaluation	Data collection method	Data collected and data type
Delivery to the clusters	1. Collation of registers from QuIP meetings (30 meetings in total across 93 hospitals) 2. Collation of VLE usage logs	1. The names, roles and hospital of each of the attendees at the QuIP cluster meetings (2 meetings per cluster) 2. The level of usage of the Virtual Learning Environment (VLE) per hospital, determined by the number of visits/views logged by any staff member from each hospital
Response of the clusters	1. Online exit questionnaire. 2. Ethnographic data	1. Free-text responses regarding the positive and negative aspects of the programme 2. Observations and interviews with key staff in the 6 ethnographic sites
Delivery at the site level – QI intervention	1. Online exit questionnaire.	1. Whether a stakeholder meeting was held (QI strategy 1) 2. Whether a QI team was formed and professional composition of any such team (QI strategy 2) 3. Whether and how data feedback occurred (QI strategy 3) 4. Whether run-charts were used (QI strategy 4) 5. Whether the patient pathway was segmented (QI strategy 5) 6. Whether the PDSA approach was used (QI strategy 6)
Response of the sites/individuals	1. Online exit questionnaire. 2. Ethnographic data	1. Free-text responses to 2 reflective questions: If you were to be involved in EPOCH again, (a) ‘what would you continue doing’ and (b) ‘what would you do differently’? 2. Observations and interviews with key staff in the 6 ethnographic sites

*QuIP* Quality Improvement Programme, *VLE* Virtual Learning Environment, *NELA* National Emergency Laparotomy Audit

### Data analysis

The programme activity and questionnaire data were analysed and reported using descriptive statistics (frequency (%) for categorical data or median (range) for continuous data). Answers to three free-text questions within the questionnaire, designed to stimulate reflection on participation in the QI programme and on leading quality improvement locally, were analysed using deductive and inductive content analysis [29]. Data were initially managed in Microsoft Excel and coded manually. Two authors (TS and TA) independently generated codes and categories emerging from these data inductively. These were compared and refined through rounds of discussion and sense-making. A set of overarching sub-themes was agreed and used these as a framework for further, more deductive, coding. Finally, these sub-themes were grouped into high-level themes for each question [29, 30]. Themes were discussed with the EPOCH ethnographic team in order to enhance validity and to support the analysis and emerging conclusions; this occurred after analysis of the ethnographic data had been completed but prior to findings being reported to the main trial team. Data analysis for the ethnographic data was based on the constant comparative method [31] and informed by sensitising concepts from the literature (for example, the role of context on QI projects) and discussions within the EPOCH team. Data from different sources, as outlined in Table 3, were analysed separately and then integrated to meet the evaluation aims for this paper. Data analysis from the questionnaire provided a cohort-wide picture of response to the programme and of intervention delivery at site level, with ethnographic data analysis adding granular detail and understanding. Integration was achieved through discussion amongst the authors responsible for analysis of the different components, identifying points of confluence and apparent contradiction between the data, and particularly focusing on the ways in which insights derived from the ethnographic work might explain or add detail to findings from the survey.

## Results

### Effectiveness and main trial outcomes

The main trial primary outcome measure was 90-day mortality. Secondary outcome measures were 180-day mortality, length of stay and hospital readmission. The stepped-wedge design allows hospitals to function as their own controls, with roughly half of the 16,000 recruited patients treated before the QI intervention, and half in hospitals activated to the intervention. The main analysis in the EPOCH trial showed no improvements in any of the primary or secondary outcomes [32].

### Process evaluation and ethnographic findings

Fifteen geographic clusters underwent randomisation including 97 NHS hospitals. Four hospitals withdrew before the start of the trial, leaving 93 participating. Programme activity data, as defined in Table 3, were available for all hospitals. Eighty-three percent (77/93) of QI leads completed the exit questionnaire. In 17/77 (22%) questionnaire returns, two or more professionals submitted a joint response. In the remainder of returns, responses were from a single QI lead. All but four responses (73/77) were from, or included input from, QI leads from the disciplines of anaesthesia or critical care. In comparison, 17/77 (22%) of responses included surgical input and 6/77 (8%) included nurse input. Across all six sites in the ethnographic sub-study, a total of 54 interviews were undertaken, with over 200 h of observation. The evaluation results are structured using the following framework: delivery of the intervention at the cluster level, response to the intervention at the cluster level, delivery of the intervention at the site level and the response to the intervention by individuals targeted (the EPOCH QI leads) [24].

### Delivery of the intervention at the cluster level

**Fig. 2:**
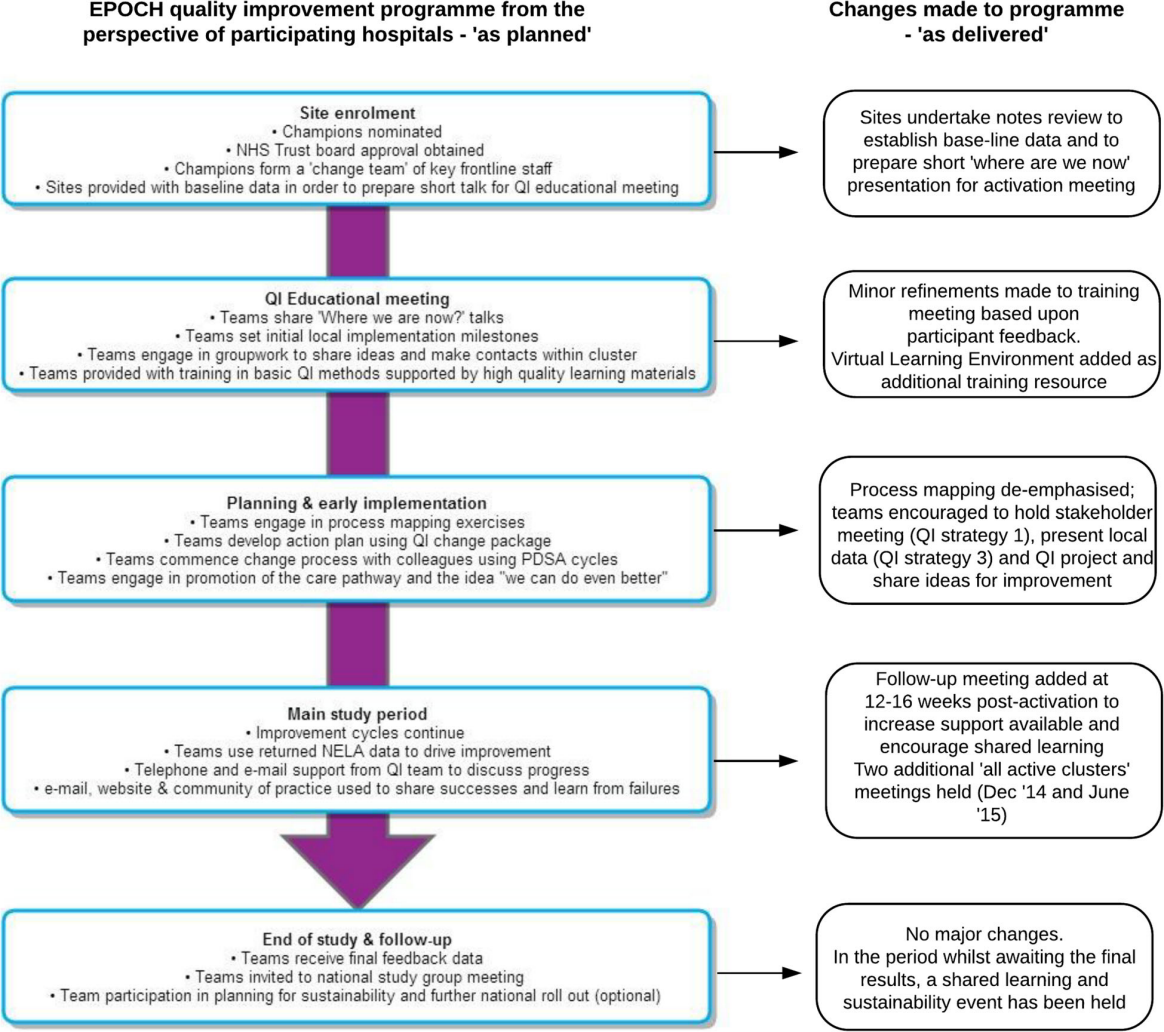
The EPOCH trial quality improvement programme. Legend: QI, quality improvement; PDSA, Plan-Do-Study-Act cycles, a specific approach to QI; NELA, National Emergency Laparotomy Audit; NHS, National Health Service

**Fig. 3:**
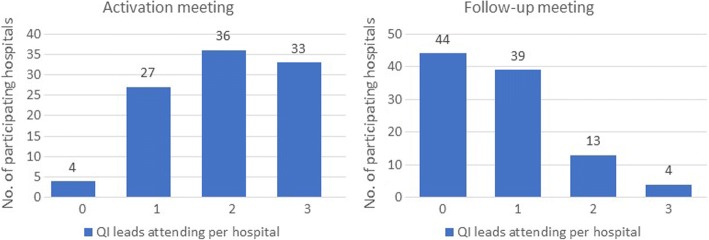
QI lead attendance at QI programme meetings

A total of 15 face-to-face, 1 day, cluster activation meetings (including QI training), planned to coincide with cluster activation, and 15 follow-up meetings (one for each geographical cluster) were held as part of the QI programme. Figure 2 summarises the EPOCH QI programme ‘as planned’ and ‘as delivered’; the major change to the plan was the addition of follow-up cluster meetings at 12–16 weeks post-activation to the intervention. Aside from local QI leads (surgeons, anaesthetists and critical care physicians), research nurses, theatre nurses and trainees in surgery and anaesthesia were the most common groups to participate in the activation meetings. The number of participants from each hospital at the follow-up cluster meeting was substantially fewer than at the first meeting. Figure 3 displays the numbers of QI leads attending the meetings from each hospital. The median number of participants (both QI leads and other invited colleagues) at the activation meetings and follow-up meetings were three per hospital (range 0–19) and one per hospital (range 0–8) respectively. The web-based resources were housed within a Virtual Learning Environment (VLE) which contained a total of 66 pages or resources, to be viewed online or downloaded, at the commencement of the programme, increasing to 84 pages or resources by the end of the study. The site could only be accessed by registered EPOCH trial local QI team members. In total, 16,120 ‘hits’ (visits to the site, page view and resource views or downloads) were logged over the course of the trial period. The median number of Virtual Learning Environment hits per hospital was 136 (min 11, max 519; IQR = 123). The number of users per hospital ranged from one to seven with a median of three users.

### Response to the intervention at the cluster level

Themes derived from responses to a free-text question in the exit questionnaire about the improvement programme are described in Table 4. Findings from the ethnographic evaluation mirror the themes described in Table 4, indicating that participants had a positive perception of the EPOCH cluster activation meetings, as well as the 12-week follow-up meetings. They felt that the EPOCH QI team demonstrated the relevance of the project and felt energised by the meetings. They also reflected positively on the practical nature of the meetings, the opportunity to share ideas and learn from others and the utility of the web-based resources and tools to analyse NELA data. Analysis of the ethnographic data indicated that buy-in from QI leads was often already high and many had achieved local improvements relevant to EPOCH’s mission long before the activation meetings. Nonetheless, even for those individuals, the activation meeting was an important place for learning and sharing experiences. It was important for local enthusiasts to see that they ‘were not alone’ in struggling to improve peri-operative care and learn how other sites managed to change aspects of care. However, themes derived from the questionnaire data indicate that satisfaction with the QI tools was more mixed, in particular the run charts to support data analysis and visualisation and the guidance on how to improve care in line with care pathway.

**Table 4:** Common themes identified from feedback regarding the Quality Improvement (QI) programme

“What was most helpful about the QI programme” (from 56 free-text responses)	“What could have been better about the QI programme” (from 36 free-text responses)
QI training (at the meetings) and online resources (*n* = 14)	More clarity about the intervention and how to implement it (*n* = 10)
Networking with colleagues from other hospitals (facilitated by meetings) (*n* = 11)	More meetings, and more input from the central team (*n* = 8)
Good communication and support (*n* = 12)	Better support / better run-chart tool (*n* = 7)
The Excel tool to generate run-charts from National Emergency Laparotomy Audit (NELA) data (*n* = 11)	A longer intervention period for those activated late (due to the stepped wedge trial design) (*n* = 7)
Enthusiasm and motivation generated by the EPOCH team and project overall (*n* = 8)	Less components in the clinical pathway (*n* = 4)

### Delivery of the intervention at the site level

**Fig. 4:**
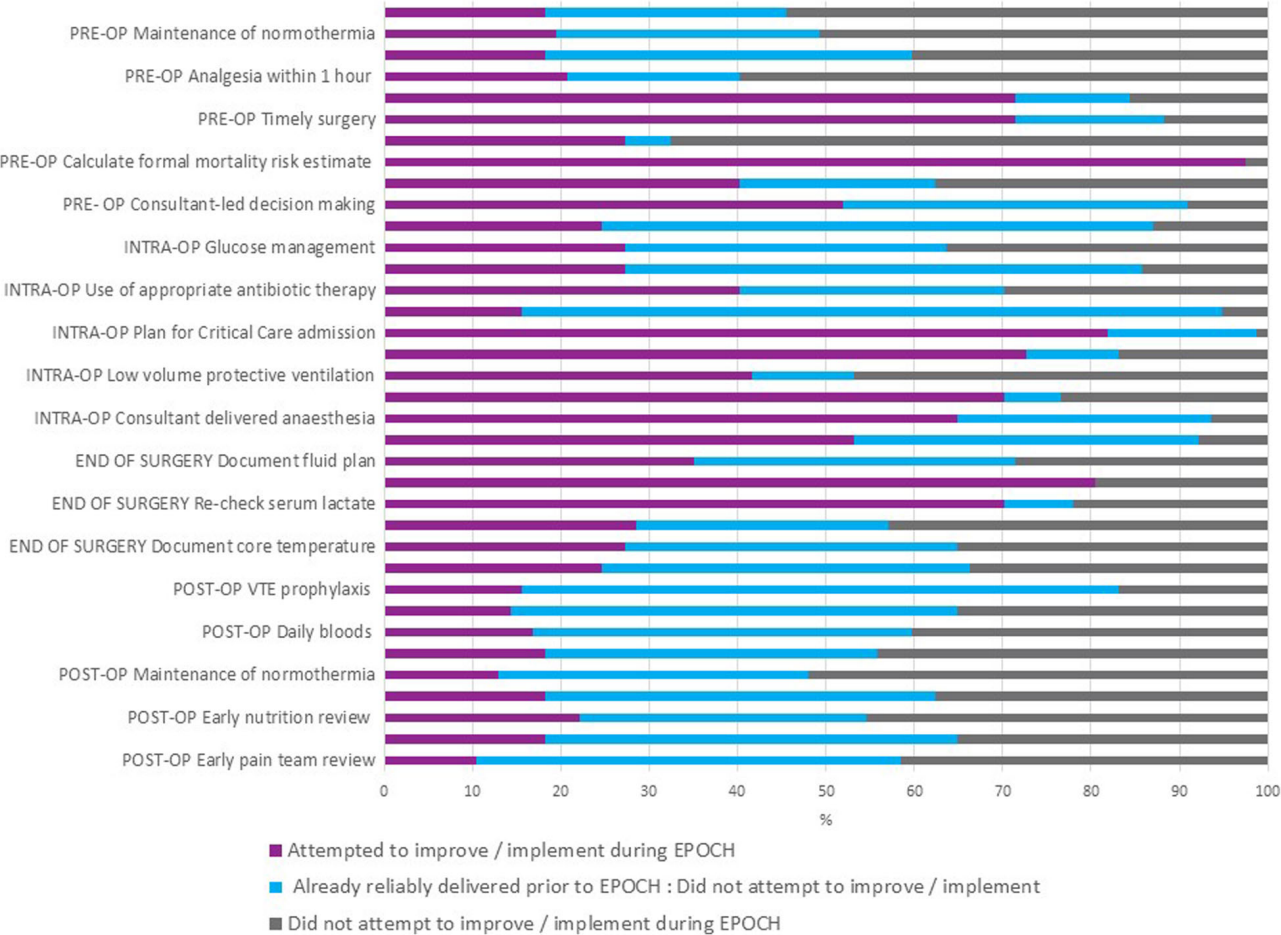
Clinical processes focussed on by hospital teams during EPOCH. Legend: CT, computer-aided tomography; WHO, World Health Organization; VTE, venous thrombo-embolism

The clinical intervention was a 37-component care pathway (see Fig. 1). Questionnaire data showed that only 11 care processes were the focus of improvement efforts in > 50% of responding hospitals; the remaining pathway components had more variable uptake (see Fig. 4 and Segmentation section below). The QI intervention comprised six strategies (see Tables 1 and 2). Questionnaire data showed that 10/77 (13%) of QI leads responding said that all six strategies had been used, 23/77 (30%) indicated five had been used, 21/77 (27%) indicated four had been used, 8/77 (10%) used three strategies, 10/77 (13%) used two and 5/77 (6%) just one. No QI lead reported zero quality improvement strategy usage. Table 5 shows the reported usage of each QI strategy. Below, questionnaire and ethnographic data are combined to elaborate on the usage of each of these strategies and the effects of these on care pathway implementation.

**Table 5:** Reported usage of each quality improvement (QI) strategy

Question related to QI strategy usage	Response (*n* = variable)
PDSA approach Did you or your colleagues use the ‘Plan Do Study Act’ (PDSA) cycle approach during your QI activities?	• 61% (45/74): Yes, sometimes • 5% (4/74): Yes, often • 34% (25/74): No
QI team formation At your site, was a formal team created to work on QI activities related to EPOCH? Definition of QI Team: A group of individuals that work together on the QI project. The team is defined by their shared goals and mutual accountability for the QI	• 60% (46/77): Yes • 27% (21/77): No • 13% (10/77): Other (comments indicated informal teams often existed)
Data collection and analysis After starting EPOCH did you or your colleagues download and analyse your local NELA data? If yes, how frequently did you do this? If yes, did you use run-charts? Were systems set up to collect National Emergency Laparotomy Audit (NELA) data prospectively?	• 79% (61/77): Yes • 21% (16/77): No • 43% (26/61): Analysing data monthly or bi-monthly • 57% (35/61): Analysing data less frequently • 92% (56/61): Used run-charts to analyse data • 51% (38/74): Yes • 49% (36/74): No
Stakeholder meeting Did you hold a stakeholder meeting as one of your QI activities? For example, a meeting for all professionals involved in patient care	• 55% (41/75): Yes • 45% (34/75): No
Pathway segmentation Please indicate statement most closely fits your hospitals improvement or implementation activity during EPOCH	• 22% (17/77): We introduced a single pathway of care (across pre-, intra- and post-operative phases) • 32% (25/77): We introduced separate pathways or care bundles for the peri-op phases • 40% (31/77): We focused on introducing individual/separate interventions • 5% (4/77): Other

### Use of Plan-Do-Study-Act

At activation meetings, the use of PDSA cycles was presented to participating teams explicitly as a model for experimentation and the planning of change, with instructions and supporting tools for putting it into practice. The data in Table 5 indicates this approach was used, but perhaps not in the regular, methodical manner recommended. The ethnographic findings also indicated that no site applied the formal PDSA methodology ‘by the book’. However, this did not mean sites failed to engage in creative experimentation. Instead, sites adopted a less formal planning approach, which included the general tenets of trying out small tests, reviewing and making further change, but typically excluding the setting of numerical goals against which to measure progress:

The only thing is we are not being particularly good at is the PDSA cycle but then again […] Well I suppose we are. We are just not doing it formally […] I have carried on and done it in a way that works and makes sense to me. (Intensive Care Consultant, Site 6)

### Team approach

At the activation meetings, QI leads were strongly advised to recruit a formal team of ‘willing’ inter-professional colleagues to work with them on local improvement activities. The data in Table 5 indicate that just under two thirds of sites had a formal team to work on this major project. All sites had committed to an inter-professional team approach by formally nominating representatives from surgery, anaesthesia and critical care; for those who managed to recruit others to their team, the benefits were apparent:

I mean the really important thing was that we had a group, from our point of view, I’ve got an engaged surgeon who I work with, and I’ve got some good junior guys, and we’ve got plenty of people who’ve actually just taken the ball and run with it […] So possibly we should be involving others but the small team we have at the moment has been quite productive and we seem to be hitting most of the QI targets with the team we have got. (Intensive Care Consultant, Site 5)

However, only three of the six sites included in the ethnographic work maintained surgical leadership throughout the intervention period; in two sites, surgical involvement in the QI team decreased after activation, and in the other site surgical involvement did not become apparent until later on. Unsurprisingly, in these sites, lack of a surgical QI lead was seen as a disadvantage to wider surgical involvement with the improvement work (see also the ‘Engagement’ section below):

It started as an anaesthetic project basically but it is really a surgery thing. […] Looking back I wish we took advantage of [having an engaged surgical lead] right at the beginning. I think we would have got more involvement with the surgeons which is obvious because they are the thing that runs right through it all. (Research Nurse, Site 1)

### Use of data feedback and run-charts

At the activation meetings, use of NELA data as a driver for engaging colleagues and monitoring improvement was promoted and tools designed for the EPOCH project were provided to do this. The data in Table 5 show that most, but not all, teams analysed their NELA data occasionally, but far fewer were doing this on a regular (monthly/bi-monthly) basis. Many sites reported challenges in simply collecting the data; only half of questionnaire respondents indicated that systems had been set up by the end of the EPOCH study to collect NELA audit data prospectively. For the other half of respondents, it was reported data collection usually involved the NELA lead (often also an EPOCH QI lead) collecting and entering data retrospectively:

We need to look at the recent outcome of the NELA. But we haven’t, because we were concentrating on NELA [data collection] and less on the EPOCH care pathway, we haven’t been able to monitor that unfortunately. (Research Nurse, Site 1)

The ethnographic findings indicated that all six sites tried hard to collect and use data in their improvement efforts. However, this was undertaken more consistently in three of the six sites. During the implementation process, the EPOCH teams that seemed more successful with data collection were also those that appeared to have achieved stronger engagement with colleagues (see section below also). This perhaps reflects the challenges of collecting the large NELA data set before any analysis, or improvement activities based upon it, could occur:

Well there is a nominal person in charge [of the NELA audit] but in terms of actual, the whole thing is devolved back round to the anaesthetic department. Well we try and get everything done, as far as possible, doing it in the operating theatre to engage the surgeons, as part of that process. Even if they only do data entry on one page, or even if we only discuss it, and one of us will do the data entry*.* (Intensive Care Consultant, Site 3)

### Engagement

At 5 weeks before activation to the intervention, sites were contacted and asked to start planning a stakeholder meeting, to coincide with activation and to engage relevant colleagues with the aims of the trial intervention and the required improvements. Just over half the respondents indicated they had held such a meeting (see Table 5). Of the 71 QI leads who responded to a question about senior support during the trial, only 15 (21%) described active executive board support for the quality improvement work related to EPOCH (e.g. funding staff time to support the project or making the project a board-level quality and safety priority). The ethnographic study allowed observation of the ongoing engagement activities that occurred beyond the initial EPOCH meetings. When local teams drew on wider connections, this appeared to work to their advantage, pulling in contacts in management, other disciplines such as radiology, and clinicians and administrators with responsibilities relevant to the pathway, for example sepsis identification and treatment. The ability to engage colleagues successfully, and encourage active involvement in improvement efforts, seemed to depend to a large extent on existing relationships:

I think, you know, we’re fairly cohesive, we have a cohesive department, and we’re not perfect, but we do. We don’t have any personality clashes that get in the way of this at the moment…We’ve had no problem with the surgical engagement and have had no problem with the anaesthetic engagement either. (Intensive Care Consultant, Site 5)

Even in sites where engagement per se was not seen to be a problem, the simple factor of the time required to have the required discussions with colleagues was raised as an issue:

I think a longer period of time would have helped because most of these changes are by default, sort of long term changes, but also there is a lot of discussion involved with them all and getting a lot of people to agree and of course each of those conversations, despite the fact that you think it is going to be quick, ends up going back to someone else and then a week passes and another week passes and before you know it a month and a half has gone and you have finally got to the conversation you wanted to have in the first place. (Consultant Anaesthetist, Site 6)

### Segmenting the pathway and decisions about the clinical pathway components

At the activation meeting, QI leads were advised to consider segmenting the proposed pathway to make the workload of implementation more manageable. Advice was offered regarding selecting which elements of the pathway to work on first and how to plan a step-wise implementation of the pathway that would work in their local context. However, by the end of the intervention period, only a fifth of questionnaire respondents (17/77) said that they had attempted full pathway intervention. Of the potential 37 pathway components, there were 11 interventions which > 50% of respondents said had been the focus of improvement efforts (Fig. 4). Eight of these 11 processes were also those captured by NELA and were the same as the main EPOCH trial process measures.

The ethnographic analysis suggests that agreement on the need for a pathway for this patient group was strong amongst QI leads and colleagues. Implementation challenges were predicted however which shaped decisions about the initial focus for improvement. These decisions were made as pragmatic choices, based on a tension between what was felt to be most important to improve versus what was manageable within work constraints:

…the surgeons and the anaesthetists and [the PI], they picked what they thought would be their top ten [from the EPOCH pathway] that we would want to institute because we thought if we tried to introduce all 30 in one go, the resistance that we would be up against would be quite difficult […] so we picked what we thought were the most important ones (Surgical Trainee, Site 4)

The idea of a step-wise approach resonated with teams, with the hope that initial success would pave the way for further pathway components to be addressed:

The ideal that we are aiming for would be to have all of the 37 (pathway) points done consistently for everybody…although the way that I think we have approached it is to cater for the ones that are perhaps easier to understand and implement…then on the back of those introduce the rest of them. (Anaesthetic SHO, Site 1)

Some other decisions came down to components of the pathway being seen as having more marginal benefits by some QI leads:

I think there were some bits that we talked about before about the inter-operative delivery so things like how you ventilate people and things like that that we didn’t necessarily want to have the argument about […] we might cross that bridge later but that wasn’t one of our first aims (Consultant Anaesthetist, Site 6)

As mentioned above, where teams did not include all clinical leads in equal leadership roles, decisions about which processes to improve often depended on which discipline was most active in the EPOCH team.

This step-wise, segmentation approach was not universally adopted however:

[The] endpoint is reduced mortality and reduced morbidity for emergency laparotomy patients. My view would be, look, we really don’t know, just do the whole bloomin’ lot and then see what happens. (Consultant Surgeon, Site 2)

In this site, their main implementation tool was thus an extensive checklist which brought the EPOCH pathway together. But by the end of the trial, they were still discussing the need to ‘implement the checklist’; progress had not been as rapid as they had hoped.

### Response the intervention by QI leads: reflections on the change process

QI leads reflected on: ‘what would you continue doing?’ and ‘what would you do differently if you were to do EPOCH again?’. 96% (74/77) of respondents left a total of 299 comments. Eighteen themes were generated for each question (36 in total) and these were further grouped into nine high-level themes (Table 6). Key themes (in terms of quantity and content of responses) include the importance of engaging colleagues (Theme 2) and some of the challenges involved in this, particularly in relation to surgical, wider inter-professional, and trainee involvement (Theme 6), and identification of robust data-collection and analysis in underpinning change (Theme 1)—and the need for more training and capacity to analyse and utilise data effectively (Theme 7). Other themes also suggest that respondents felt that much of the approach advocated by EPOCH would work (Themes 3-5), but with important challenges around capacity and persuading colleagues—whether gently or more coercively—of the need to contribute to change (Themes 8-9).

**Table 6:** Themes emerging from QI leads reflections on leading improvement

High-level themes	Sub-themes (number of supporting comments)
What QI leads would continue doing	
1. Keep working on data collection and feedback	Providing feedback on performance, incl. data feedback (30) Use run-charts (19) Good data collection process/data collection support (14) Using data to create situational awareness (4)
2. Keep working on engagement, involvement and collaboration	Engage/involve all relevant stakeholders (22) Interprofessional involvement (9) Form a QI team (8) Engage/involve trainees (4) Identify enthusiastic colleagues (4) Collaborate with other hospitals (2) Obtain senior support for the project (2)
3. Using a ‘systems thinking’ approach to improvement	Hardwire changes into system (9) Building risk scoring into care pathway (8) Use a checklist/care bundle approach (2)
4. Specific clinical interventions	Clinical interventions (9) Risk stratification (6)
5. Use an iterative approach to change	Take an incremental/stepped approach to improvement (6) Persist with implementation (2)
What QI leads would do differently	
6. Engage and involve people more effectively	Wider engagement of stakeholders (17) More surgical engagement/involvement in project (15) More interprofessional involvement (10) Better engagement/involvement of trainees (6) Form a larger QI team (5| Involve more people (3)
7. Get data collection and feedback right	Improve data collection/more data support (17) More data feedback (8) More data analysis (4)
8. Obtain stronger senior support for the project	Stronger senior leadership/board level support (16) More protected time for the project (7)
9. Work on own leadership/ project management skills	Manage the QI team more effectively (10) Get started sooner (6) Be more forceful (3) Focus on motivation/behaviour change (2) Use an iterative approach (2) More collaboration with other hospitals (2) Better planning of improvements / system changes (2)

*QI* quality improvement; *Run-chart* a specific type of time-series chart used in quality improvement

### Context

Limited resources, both human and financial, and organisational upheaval were often mentioned, in particular in Ethnographic Site 3, although it is likely that this experience was shared by a significant subset of the 93 hospitals in the trial. Across almost half the trial sites, a lack of organisational support for data collection was noted. The challenges this posed for QI leads must not be underestimated, with the burden of collecting data (for NELA and ostensibly for use as part of the EPOCH improvement work) may have overwhelmed many. As mentioned above, teams often wanted to do more but struggled to find time:

Again, it’s finding the time to do all this stuff…the trust hasn’t given anyone any time for this, so people are doing it, you know, because they want to. So, you know, it would help if it had time funded time for it, but you know that’s never going to happen in the NHS […] not at the moment. (Intensive Care Consultant, Site 5).

## Discussion

The principal finding of this process evaluation was that the QI programme delivered the QI skills training and resources as intended and the programme was generally well received by QI leads and there was an overall sense of motivation to address the challenge of high-mortality for this patient group. Local adaptation to both the QI and clinical interventions was actively encouraged, but the extent of variability and adaptation in the implementation process was greater than anticipated. There were only 11 clinical interventions which more than half of teams attempted to improve from the clinical pathway (the ‘hard core’ of the intervention) and only half of the trial cohort reported using five or all six of the QI strategies (the ‘soft periphery’ of the intervention) designed to enable pathway implementation [16]. Ethnographic findings indicated that QI leads predicted, and often experienced, multiple and often significant challenges as they attempted to lead change in their hospitals, which shaped which components of the pathway they chose to focus on first and how they approached implementation. The main trial results showed no effect of the intervention on patient outcomes or care processes. Our experience during the QI programme (meeting teams, reviewing their data) suggests that some hospitals were able to make modest, and sometimes substantial, improvements in care processes but the main trial analysis was not designed to provide this level of granularity.

When testing clinical interventions within a clinical trial, it is important to make the distinction between the design of the intervention and the operational elements required for effective delivery [33]. Our process evaluation, discussed in this paper, adds to the main trial findings by providing insight into the challenges at both the design (or programme) level and the hospital (operational) level. At the design level, adaptability is often essential in ensuring that quality improvement interventions can fit within different contexts, and this was built into the EPOCH intervention. However, fidelity to key parts of an intervention is also important to maximise likelihood of success [34]. In this case, it may have been that an intervention design that focussed on a smaller number of strategies might have achieved greater fidelity and, therefore, greater impact on patient outcomes. This may be especially relevant given that data from both the ethnography and the exit questionnaire suggest that, at the operational level, QI leads faced many local challenges including lack of engagement of colleagues and hospital executives. Even in sites where such challenges were minimal, QI leads were making choices about which clinical components of the pathway to focus on first, in recognition that implementing the entire pathway may be beyond the limited time and resources they had. Thus, the extent of the task required, combined in many sites with organisational challenges, may have meant that many teams simply ran out of time to implement the pathway within the intervention period. Earlier, smaller, studies have shown that marked improvement may take time and can continue after the intervention period [14]. Data was also an operational challenge for many. NELA had only commenced 4 months before the start of the trial; 20 months after the launch of NELA, at the end of this study, only half of hospitals reported having prospective data collection systems in place. It is likely therefore that many QI leads were focussed on collecting and in-putting data to the detriment of other improvement activity. A key theme from the reflections of QI leads was that they would have liked to have had better mechanisms not only for data collection but also for data feedback. Whilst data is central to any quality improvement project, it is the use of this data through feedback, combined with other improvement strategies, that is likely to achieve more robust results [19, 35–37]. If future QI programmes are to capitalise on concurrent national audits or other ongoing data collection, the timings need to be considered to allow embedding of data collection processes before the start of the improvement work which may take considerably longer than anticipated [35].

There are other explanations for our failure to change the primary outcome metrics. It is possible that our programme theory was incorrect, and there was only a weak causal link between the interventions and ultimate outcomes. This seems unlikely given the evidence base for the clinical and quality improvement interventions. The EPOCH trial intervention ran at a time of significant change in the British NHS, including major system re-organisation and considerable fiscal instability for many hospitals [38]. These changes may have impacted on staff morale and on the ability of clinicians to engage with and focus on their local projects [39]. Another conclusion that might reasonably be drawn from our evaluation is that the EPOCH trial intervention was too ambitious. Even where QI leads developed the capabilities to enable change (e.g. through use of the QI strategies), they were asked to lead that change in addition to their regular clinical commitments and may not have had the capacity, in terms of time, resources and other personnel, to do so. The social aspects of improvement are as likely to be as important as more technical aspects, such as data analysis and feedback, but QI leads used the social QI strategies less than those related to data. Building and maintaining effective social relationships is time-consuming and challenging, and the uptake of ‘non-technical’ and ‘socio-adaptive’ interventions can be low amongst health professionals [18]. However, a key reflection of QI leads was that they would have liked to spend more time engaging and involving colleagues. We would suggest therefore more emphasis and training in socio-adaptive interventions should be built into future programmes together with a recognition that dedicated time is required to support frontline staff in prioritising such interventions [40, 41]. Some leads reflected on their difficulties in engaging with senior or executive-level colleagues, and only a fifth of respondents indicated they received active support from their board. Effective quality improvement requires a reciprocal relationship between the employee and the organisation, and lack of organisational support is likely to have been an important barrier to improvement [42, 43]. This is an important lesson; if the goodwill and motivation of frontline staff is to be mobilised for improvement work, then adequate time and support in the workplace plus training is required to give these professional the best chance of success. This has ramifications for those designing future programmes, senior management and national-level policy makers.

In relation to the delivery of the programme, the time available to coach teams was limited in comparison with other reported quality improvement interventions, such as the Institute for Healthcare Improvement Breakthrough Series Collaborative model [44]. Our training programme was designed as a parsimonious intervention, with face-to-face meetings limited, so that it might be adapted and replicated widely if proven successful. A higher intensity programme might have led to greater intervention fidelity, although recent evidence suggests that this may not always be the case [45, 46]. EPOCH may have suffered from the lack of a pilot trial and perhaps future similar interventions should be piloted first [47], or use a cluster trial design that allows for iterative intervention development within the trial period to enable ongoing intervention optimisation [48].

A major strength of this study is that it provides a full, detailed description of how a large-scale trial of a complex intervention was designed, delivered and received, at over half the hospitals in the UK NHS. Following calls for better intervention reporting, we hope we have provided insights into possible reasons why ultimately the trial was unsuccessful and learning for future studies of this nature [23, 49]. The evaluation was conducted by researchers both inside and outside the main trial team, offering both detailed, nuanced knowledge of the trial, with an external perspective; all data collection and analysis were completed before the trial results were known. This study also has several limitations. The process evaluation relied in part on self-reported data, often collected from a single representative of each hospital. A response rate of 83% suggests that our data were largely representative of the entire EPOCH trial cohort. However, because non-responders may have had different experiences with the EPOCH programme, it is possible that some relevant factors may be missing. Self-reported data may be subject to both recall and/or social desirability bias. To minimise recall bias, we started collecting data within a month of the completion of the trial. Whilst we cannot quantify the magnitude of potential social desirability bias, many respondents reported both positive and negative experiences and many reported not using several of the quality improvement strategies. Responses tended to be relatively brief, with no possibility for respondents to elaborate on interesting or unclear statements. Thus, we found these data to be a useful adjunct to, but no substitute for, the more extensive qualitative insights provided by the ethnographic study.

## Conclusion

Programmes designed to support clinician-led improvement may need to focus on both developing the necessary QI capabilities whilst also advocating (or even mandating) clear organisational support for these professionals to lead change. Additional capacity, including job-planned time to engage stakeholders plus data support and/or adequate date collection mechanisms, are likely pre-requisites for the successful delivery of complex interventions, such as implementing a care pathway for emergency surgery.

## Additional file

Additional file 1: ᅟ(PDF 1223 kb)

## Abbreviations

EPOCH: Enhanced Peri-Operative Care for High-risk Patients
NELA: National Emergency Laparotomy Audit
NHS: National Health Service
PDSA: Plan Do Study Act
QI: Quality improvement
QuIP: Quality Improvement Programme
UK: United Kingdom
VLE: Virtual Learning Environment
