# Correction to: Improving care at scale: process evaluation of a multi-component quality improvement intervention to reduce mortality after emergency abdominal surgery (EPOCH trial)

**DOI:** 10.1186/s13012-018-0840-8

**Published:** 2018-12-10

**Authors:** T. J. Stephens, C. J. Peden, R. M. Pearse, S. E. Shaw, T. E. F. Abbott, E. L. Jones, D. Kocman, G. Martin, on behalf of the EPOCH trial group

**Affiliations:** 1 Critical Care and Perioperative Medicine Research Group, WHRI, c/o Adult Critical Care Unit, The Royal London Hospital, London, E11BB UK; 2 Keck School of Medicine, University of Southern California, Los Angeles, USA; 3 Nuffield Department of Primary Care Health Sciences, University of Oxford, Oxford, UK; 4 Warwick Clinical Trials Unit, University of Warwick, Coventry, UK; 5 SAPPHIRE Group, Department of Health Sciences, University of Leicester, Leicester, UK; 6 THIS Institute (The Healthcare Improvement Studies Institute), University of Cambridge, Cambridge, UK

**Correction to: Implementation Science (2018) 13:142**

**https://doi.org/10.1186/s13012-018-0823-9**

Following the publication of this article [1], the authors reported a number of errors which are given below.

The published article contained a discrepancy in Table 3 between the PDF and HTML versions of the article. In the PDF version Table 3 was missing a row as shown below:

**Table 3:** Data collected for process evaluation

Aspect of process evaluation	Data collection method	Data collected and data type
Delivery to the clusters	1. Collation of registers from QuIP meetings (30 meetings in total across 93 hospitals) 2. Collation of VLE usage logs	1. Free text responses regarding the positive and negative aspects of the programme 2. Observations and interviews with key staff in the 6 ethnographic sites
Delivery at the site level – QI intervention	1. Online exit questionnaire.	1. Whether a stakeholder meeting was held (QI strategy 1) 2. run-charts were used (QI strategy 4) 5. Whether the patient pathway was segmented (QI strategy 5) 6. Whether the PDSA approach was used (QI strategy 6)
Response of the sites / individuals	1. Online exit questionnaire. 2. Ethnographic data	1. Free text responses to 2 reflective questions: If you were to be involved in EPOCH again, a) ‘what would you continue doing’ and b) ‘what would you do differently’? 2. Observations and interviews with key staff in the 6 ethnographic sites

*QuIP* Quality Improvement Programme, *VLE* Virtual Learning Environment, *NELA* National Emergency Laparotomy Audit

Table 3 should in fact contain four rows as in the HTML version:

**Table 3:** Data collected for process evaluation

Aspect of process evaluation	Data collection method	Data collected and data type
Delivery to the clusters	1. Collation of registers from QuIP meetings (30 meetings in total across 93 hospitals) 2. Collation of VLE usage logs	1. The names, roles and hospital of each of the attendees at the QuIP cluster meetings (2 meetings per cluster) 2. The level of usage of the Virtual Learning Environment (VLE) per hospital, determined by the number of visits / views logged by any staff member from each hospital
Response of the clusters	1. Online exit questionnaire 2. Ethnographic data	1. Free text responses regarding the positive and negative aspects of the programme 2. Observations and interviews with key staff in the 6 ethnographic sites
Delivery at the site level – QI intervention	1. Online exit questionnaire	1. Whether a stakeholder meeting was held (QI strategy 1) 2. Whether a QI team was formed and professional composition of any such team (QI strategy 2) 3. Whether and how data feedback occurred (QI strategy 3) 4. Whether run-charts were used (QI strategy 4) 5. Whether the patient pathway was segmented (QI strategy 5) 6. Whether the PDSA approach was used (QI strategy 6)
Response of the sites / individuals	1. Online exit questionnaire 2. Ethnographic data	1. Free text responses to 2 reflective questions: If you were to be involved in EPOCH again, a) ‘what would you continue doing’ and b) ‘what would you do differently’? 2. Observations and interviews with key staff in the 6 ethnographic sites

*QuIP* Quality Improvement Programme, *VLE* Virtual Learning Environment, *NELA* National Emergency Laparotomy Audit

Furthermore, Fig. 4 was condensed upon submission, which resulted in half the data lines losing their legends in both the HTML and PDF version. Figure 4 originally appeared as follows:

**Fig. 4:**
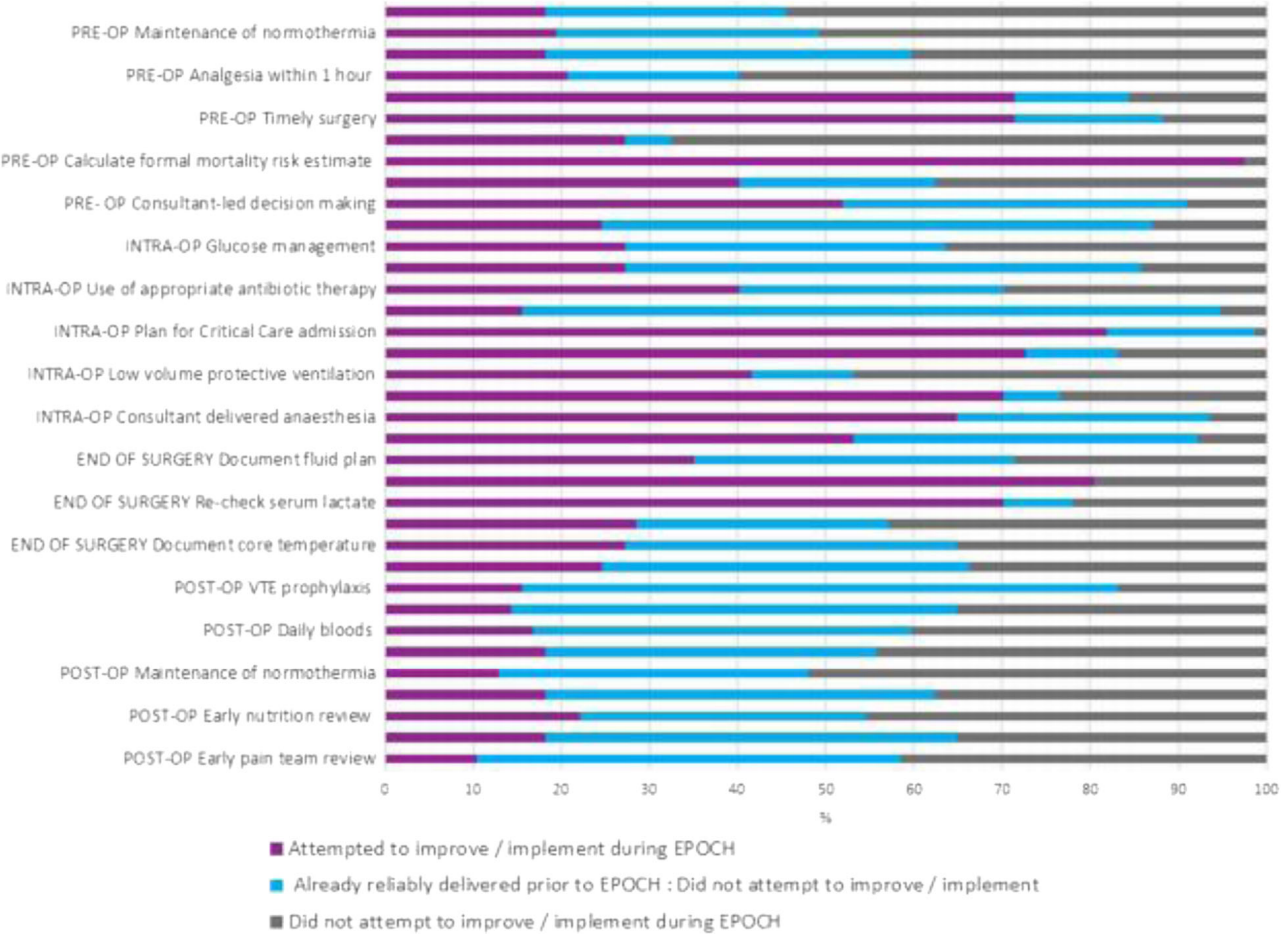
Clinical processes focussed on by hospital teams during EPOCH. Legend: CT, computer-aided tomography; WHO, World Health Organization; VTE, venous thrombo-embolism

The correct version of Fig. 4 is:

**Fig. 4:**
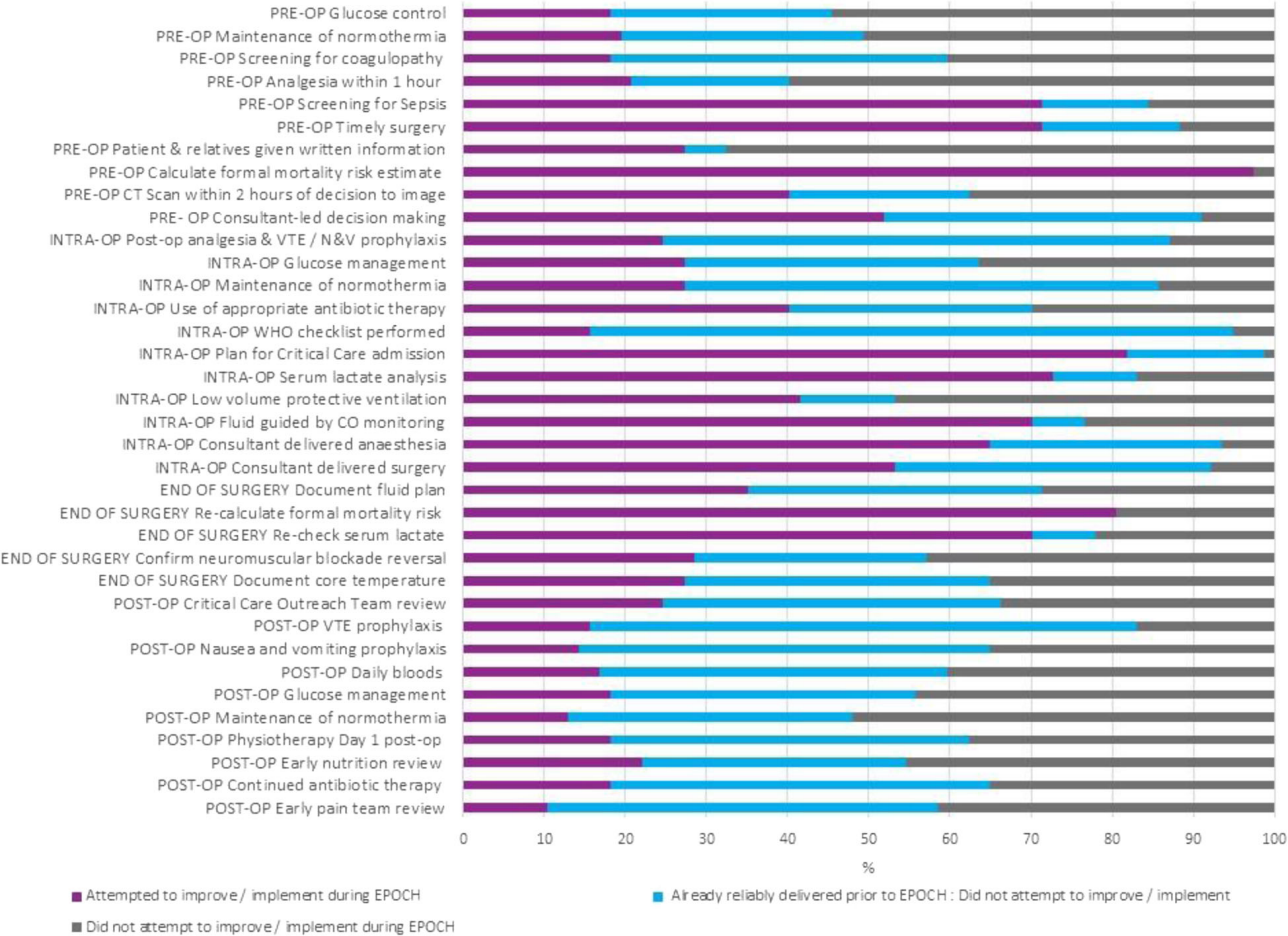
Clinical processes focussed on by hospital teams during EPOCH. Legend: CT, computer-aided tomography; WHO, World Health Organization; VTE, venous thrombo-embolism

Furthermore, the names of several members of the EPOCH trial group were not processed correctly and should have been processed as follows (Given Name, Family Name):

- Mike Bradburn
- Fiona McMenemie
- Jason Cupitt
- Robert Thompson
- Nick Harper
- Simon Sleight
- Belinda Cornforth

The authors also noticed they submitted an incorrect list of authors and the correct list is shown below:

EPOCH Trial Group:

Abdel Omer; Abhiram Sharma; Abigail Patrick; Adam Paul; Adam Wolverson; Adrian Fawcett; Adrian Jennings; Ajaya Mull; Ajit Sivasankaranand; Alan Morrison; Alastair Ankers; Alastair Rose; Alexandra Scott; Alexandra Williams; Alison Hool; Alison Pickford; Alistair Roy; Alistair Steel; Alister Myers; Almas Rehman; Amanda McCairn; Amanda Stevens; Amir Rafi; Amira Girgis; Amit Shukla; Ana Alegria; Andreas Brodbeck; Andreou Prematie; Andrew Brennan; Andrew Burtenshaw; Andrew Claxton; Andrew Lindner; Andrew Miller; Andrew Thorniley; Andrew White; Andy Thacker; Anil Hermandes; Anita Jhamatt; Anita Sugavanam; Anitha Holtham; Anjay Talwar; Anne Scase; Anthony Parsons; Arnab Bhowmick; Arnth Engel; Ash Prabhudesai; Ashok Raj; Asif Jah; Ayodele Obideyi; Babu Muthuswamy; Bala Maiya; Banwari Agarwal; Barclay Tofte; Belinda Cornforth; Beth Hale; Biju Aravind; Blenk, Karl; Britta O'Carroll-Kuehn; Broad, Dan; Bruce Gibson; Carmen Correia; Carol Mcarthur; Carolyn Way; Catherine Farrow; Catherine Harden; Catherine Jardine; Charles Knowles; Chitre Vivek; Chloe Rochester; Chris Coldwell; Chris Dawson; Chris Homer; Chris Lewis; Chris Nutt; Chris Thorn; Chris Wilson; Christine Bronder; Christopher Macklin; Clare Stapleton; Colin Pow; Craig Lyness; Craig Morris; Dale Vimalchandran; Damian Laba; Dan Freshwater-Turner; Daren Subar; David Bottomley; David Browell; David Gerrard; David Inglis; David Melville; David Monk; David Pogson; David Riddington; David Saunders; David Stanley; Davina Ross-Anderson; Dawn Hales; Dean Millican; Debbie Shaw; Denzil May; Dewi Williams; Dhiraj Ali; Diane Monkhouse; Diane Murray; Dipankar Mukherjee; Dolores Mateo; Dom Hurford; Dominic Sebastian; Donna Doyle; Edward Curtis; Edward Lams; Edyta Niebrzegowska; Elizabeth Hall; Elizabeth Harwood; Emanuel Cirstea; Emma Brennan; Emma Davis; Emma Durant; Emma Leno; Erin Mcilveen; Essam El-Damatty; Esther Cook; Ewen Griffiths; Ewen Harrison; Faisal Baig; Fanus Dreyer; Fenella Welsh; Fiona McMenemie; Flavia Menezes; Flora Bailey; Fran Haigh; Frances Mosley; Francesca Rubulotta; Frankie Dorman; Gabriele Marangoni; Gail Stark; Gail Williams; Gareth Moncaster; Gary Minto; Gavin Bryce; Geoff Watson; Georgia Knight; Gethin Williams; Gillian O'Connell; Giovanni Brescia; Glen Arnold; Gordon Milne; Graham Wilson; Grainne O’Dwyer; Grant Sanders; Greg Lawton; Gudrun Kunst; Guy Finch; Guy Nash; Guy Rousseau; Hamish Noble; Hannah Smith; Harjeet Narula; Hazel Stuart; Heather Pratt; Helen Agostini; Helen Black; Helen Howes; Helen Langton; Helen Porter; Helena Stafford; Hitesh Patel; Huw Davis; Iain Christie; Ian Clement; Ilona Raulusaite; Inga Misane; Ingeborg Welters; Isabella Karat; Jack Carmichael; Jack Parry Jones; Jagdeep Singh; James Bromilow; James Brown; James Burrow; James Harris; James Kirby-Bott; James Limb; Jamie Greenwood; Jane Blazeby; Janindra Warusavitarne; Jason Cupitt; Jay Gokhale; Jay Susarla; Jennifer Edwards; Jennifer Spimpolo; Jenny McLachan; Jenny Ritzema; Jenny Wright; Jens Full; Jeremy Marwick; Jessica Thrush; John Abercrombie; John Corson; John Griffith; John Hopper; John Mackinnon; John Tansley; Jonathan Mccullough; Jonathan Paddle; Jonathon Barker; Jonathon Mole; Jonny Wilkinson; Josef Watfah; Jost Mullenheim; Julian Sonksen; Julian Stone; Julie Colley; Julie Furneval; Julie Wakeford; Julie Wollaston; Justin Woods; Jyrki Karvonen; Kaighan Lynne; Kamal Aryal; Kar​thik​ Surendran; Karan​ Verma; Karen Burns; Karen Simeson; Karvonen Jyrki; Kate Wong; Kathryn Cain; Kathryn Gill; Katie Cooke; Keiarash Jovestani; Kenneth Adegoke; Kevin Rooney; Kevin Sim; Khaled Razouk; Kim Jemmet; Kirosh ​Shankar; Kirsty Baillie; Kirsty Everingham; Krishnamurthy Somasekar; Kumar Panikkar; Lampros Liasis; Laura Graham; Laura Rooney; Lawrence Wilson; Lee Baldwin; Leilani Cabreros; Liam​ Hudson; Linda Graham; Lindsay Bailey; Lorna Burrows; Louise Bell; Lynn Stewart; Lynn Taylor; Lynsey Kightly; Magdy Khater; Maitra Ishaan; Majed Al Shama; Makvana Sonia; Malcolm Sim; Malcolm Watters; Manab Mohanty; Mansoor Akhtar; Mansoor Sange; Marcus Wood; Maria Bews-Hair; Maria Lawson; Marion Obichere; Mark Blunt; Mark Cartmell; Mark Coleman; Mark Henwood; Mark Munro; Mark Pulletz; Mark Snazelle; Mark Watson; Mark Wilkinson; Marta Campbell; Marta Januszewska; Martin Leuwer; Martin Northey; Martin Stotz; Martyn Cain; Massimo Varcada; Matt Gardner; Matt Outram; Matthew Gaughan; Matthew Tutton; Maurizio Cecconi; Meghna Sharma; Melanie Tan; Michael Chadwick; Michael Crabtree; Michael Gillies; Michael Karlikowski; Michael Machesney; Michael Martin; Mike Bradburn; Mike Gay; Nabil El-Masry; Nabua Gerstina; Nada Hadi; Nandita Divekar; Nat Natarajan; Natalie Dickinson; Nathan Pushpa; Nathan Borgeaud; Nazar Abdul; Nazzia Mirza; Neil Cruickshank; Neil Flint; Neil Kukreja; Nicholas Watson; Nick Bunker; Nick Harper; Nick Mason; Nicola Cook; Nicola Lythell; Nicola Radford; Nicola Stanix; Nicole Robin; Nigel Hollister; Nigel Suggett; Niko Van De Velde; Nikolaos Makris; Olga Tucker; Oliver Hill; Oliver Zuzan; Oluremi Odejinmi; Otto Mohr; Paddy Collins; Panna Patel; Paul Harrison; Paul Mclaren; Paul O'Loughlin; Paul Ziprin; Paul Noble; Pedro Cunha; Peeyush Kumar; Peter Alexander; Peter Chan; Peter Davies; Peter Fitzgerald; Peter Lamb; Peter Richardson; Phil Dodd; Phil Hopkins; Phillippa Pemberton; Phoebe Bodger; Pieter Bothma; Piroska Toth-Tarsoly; Preeti Kuduvalli; Qamar Iqbal; Rachael Craven; Rai Kuldip; Raj Patel; Rajesh Dumpala; Raman Guruswamy; Ramani Moonesinghe; Rame Sunthareswaran; Ramesh Rajagopal; Ranjit Ganepola; Raoul Benlloch; Razeen Mahroof; Rich Gibbs; Richard Hartopp; Richard Haslop; Richard Howard-Griffin; Richard Morgan; Richard Pugh; Richard Wharton; Ricky Lewis; Rob Chambers; Robert DeBrunner; Robert Shawcross; Roger Townsend; Roshan Lal; Rovan D'souza; Rowena Felipe; Roy Fernandes; Ruth Griffin; Ruth Thomas; Sally Roth; Sam Andrews; Sam Waddy; Samer Doughan; Sami Farhat; Sami Ijaz; Sandeep Varma; Sanjay Wijeykoon; Sara Pick; Sarada Gurung; Sarah Beavis; Sarah Bowery; Sarah Buckley; Sarah Downey; Saravanna Sagadai; Satish Singh; Savvas Papagrigoriadis; Sean Cope; Sean McAfee; Sean Mcmullan; Senthil Nadarajavan; Sergei Vaganov; Shameem Sarfi; Sheshagiri Bengeri; Shrisha Shenoy; Shubha Vashisht; Sian Bhardwaj; Sid Riddington; Simon Bailey; Simon Fletcher; Simon Gibson; Simon Harris; Simon Hester; Simon Parrington; Simon Sleight; Simon Smart; Singh Gursevak; Somi Desikan; Sophie Noblett; Stacy Hodges; Stas Janokowski; Stefan Pulsa; Stelios Chatzimichail; Stella Vig; Stephanie Bell; Stephen Baxter; Stephen Harris; Stephen Lake; Steve Fletcher; Steve Hutchinson; Steven Henderson; Stewart Prestwich; Stuart Mercer; Sudha Garg; Surjait Singh; Susan Dowling; Susan Jain; Susan Moug; Susan Tyson; Susie Baker; Syed Iftikhar; Tabitha Tanqueray; Taj Saran; Tamas Szakmany; Tamsin Rope; Tamzin Cuming; Tanuja Shah; Tariq Hussein; Tezas Stergios; Therese Murray; Thomas Evans; Thomas Medici; Thomas Parker; Tim Campbell-Smith; Tim Geary; Tim Harvard; Tim White; Tom Abbott; Tom Edwards; Tom Morgan-Jones; Tom Owen; Tomas Jovaisa; Una McNelis; Valerie Hilton; Vamsi Velchuru; Vanessa Linnett; Vanessa Tucker; Veena Naik; Victoria Banks; Vishal Patil; Vivek Chitre; Vlad Kushakovsky; Wael Khalaf; Wayne Wrathall; Will Brady; Xavier Escofet; Yasser Mohsen; Ying Hu.

The original article has been corrected. The publisher apologizes to the readers and the authors for any inconvenience caused by these errors.
