# Supplementary material for: Improving care at scale: process evaluation of a multi-component quality improvement intervention to reduce mortality after emergency abdominal surgery (EPOCH trial)
Source: Implement Sci. 2018 Nov 13;13:142. doi: 10.1186/s13012-018-0823-9 (PMC6233578; doi:10.1186/s13012-018-0823-9)
Supplement: Supplementary file 1 — ᅟ(PDF 1223 kb) [file 13012_2018_823_MOESM1_ESM.pdf]

Details of EPOCH Quality Improvement Programme delivery

Nominated QI Leads were informed 14 weeks before the date of activation to the quality improvement intervention. Five weeks before activation QI leads were sent a 'pre-activation' checklist (see below), which included the requirement to review five sets of notes from recent patients to establish current performance and identify gaps in care delivery. A notes review tool was provided (see below), and each hospital presented their findings at the initial cluster meeting. The nominated QI leads at each hospital were the conduit through which the EPOCH quality improvement intervention, and therefore the improvements in care, were delivered. A training package was designed for hospital QI Leads and their colleagues, the main content of which was delivered at an initial one-day cluster activation and training meeting (see below). The training was designed specifically to support the uptake of the six quality improvement strategies (Table 1) that would enable the translation of the programme theory into practice, and employed a mixture of didactic, workshop and discussion sessions. Publicity resources, such as pens, lanyards and mugs were distributed to each team on the day, to be shared with colleagues to raise awareness about participation in the EPOCH study.

A Virtual Learning Environment (VLE) housed all training resources and acted as a repository for all the tools and documents required to enact the EPOCH quality improvement strategies. This was created to support QI leads who had attended the training and desired further quality improvement resources, as well as ensuring that QI leads and other team members who could not attend the training meeting could view all the necessary presentations and resources. In particular, the site housed a tool developed to allow the creation of time-series charts, using local NELA data, to allow QI leads to monitor key care processes during the improvement period. It also incorporated an interactive 'route-map', providing evidence sheets for each of the clinical recommendations within the EPOCH pathway (See below). All hospital QI leads were automatically registered for the VLE five weeks prior to activation and could request additional colleagues and team members to be registered.

Once a cluster was activated, telephone and email support for the intervention was available. Separate email contact, including a regular newsletter, was maintained with all hospitals (both activated and those in-waiting) by the trial manager. Each hospital was offered a small amount of funding (£3700 GBP) for QI leads to spend on relevant activities. Half-day follow-up meetings (see below) were added soon after commencement of the study, to offer teams formal opportunities to share successes and challenges as they progressed, supported by advice from the programme leads. All clusters were offered a follow-up meeting. Two further, optional, large-scale meetings were held (in December 2014, June 2015) to allow all activated teams to come to together to learn more about quality improvement and each other's successes and challenges (see below). These meetings employed a mixture of didactic, workshop and discussion sessions and were designed to be both motivational and educational. A shared learning and sustainability event was held in March 2016 after the intervention period had ended and the trial was in follow-up, to support teams in their on-going quality improvement work.

Example of page from EPOCH Virtual Learning Environment, with pre-activation instructions

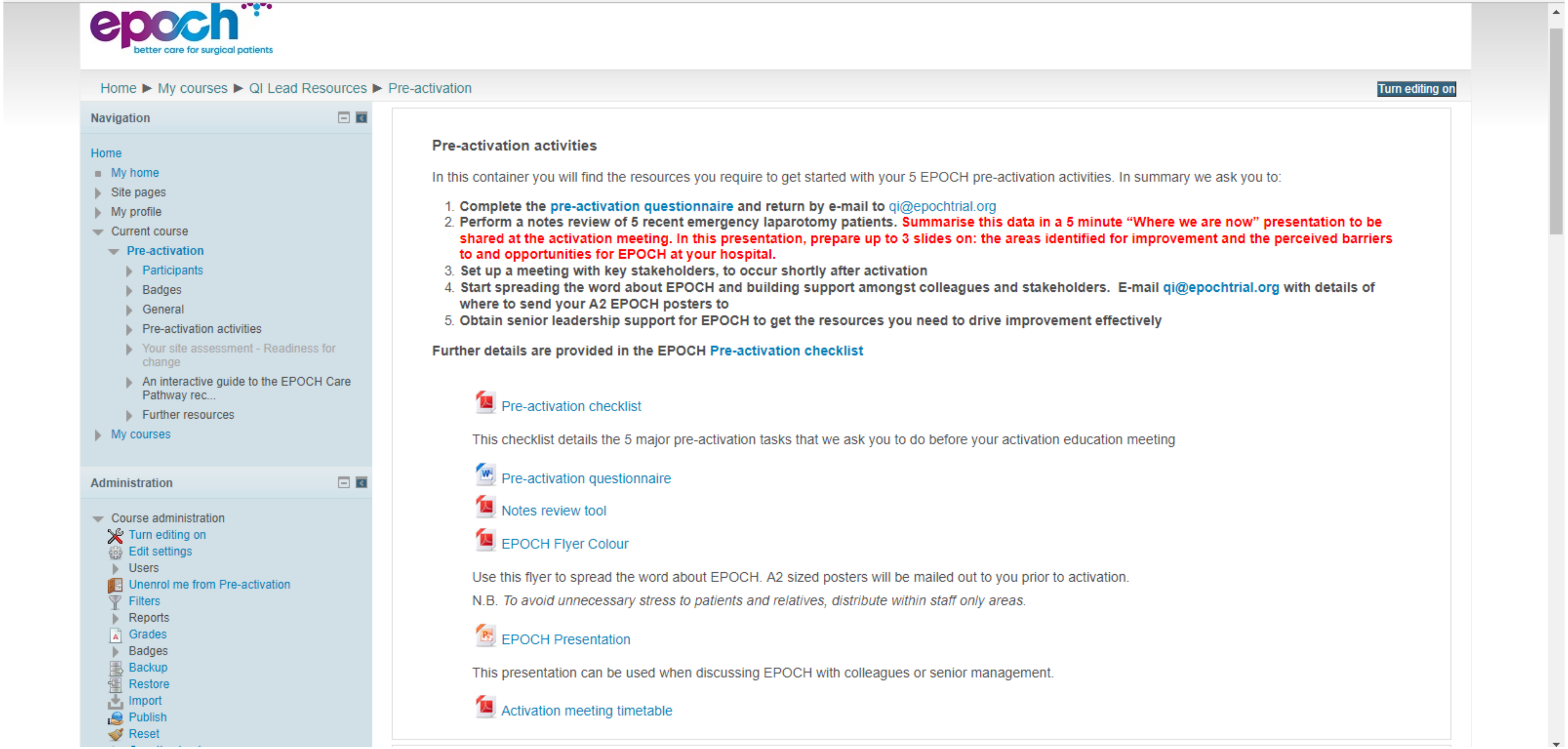

Timetable - Cluster activation and training meeting

Time	Duration	Session	Speaker / Workshop Lead	Participant resources to take away	Notes
9.30	10.00	• Coffee<br>• Registration<br>• Complete pre-course questionnaires			Questionnaires
10.00	15	• Introductions and learning objectives for the session.			
10.15	15	*EPOCH - brief overview of the trial*<br>• Trial design, outcomes etc			
10.30	60	*Site presentations: "Where we are now"*<br>• Each site presents data on current performance	Participants		
**11.30**	15	*Coffee*			
11.45	20	*Presentation: ELPQUIC – experience and results of implementation*<br>• Carol presents The Emergency Laparotomy Quality Improvement Collaborative (ELPQuIC) results	Carol Peden	Available on EPOCH VLE	
12.05	30	*Q&A: The EPOCH pathway & recommendations*<br>• Q&A on the recommendations, using the routemap to clarify any concerns or doubts	Mandeep Phull-Kerr/Tim Stephens	Available on EPOCH VLE	Routemap hardcopy
12.35	15	*Workshop: Segmentation*<br>• Teams brainstorm what will be initial improvement area<br>○ 3 - 4 areas and 3 steps toward change for each<br>• Teams will feed back ideas after lunch		An 'action plan' for weeks 1-4	Scan / photograph these and refer back to them at follow-up meeting
12.50	30	Lunch			
13.20	10	Outline of how to improve: (5 mins)<br>1. Data<br>2. Engagement	Tim Stephens / Carol Peden		

		3. Testing changes			
13.30	20	*Presentation: Data for Improvement presentation*	Carol Peden	Available on EPOCH VLE	
13.50	30	*Workshop: Data for Improvement*<br>• Show group run-chart maker<br>• Go through data to be extracted from NELA dataset<br>• Demonstrate how to use the run-chart maker<br>• Discuss how best to share data to drive improvement	Tim Stephens	Pre-made Excel workbook available for participants on EPOCH VLE	
14.20	20	*Activity: The Improvement Cycle*			Need spare paper for exercise
14.40	20	*Presentation: The Improvement Cycle*<br>• This session will provide an overview of and scientific basis for the improvement cycle and explain why it is at the heart of QI methodology	Carol Peden	Available on EPOCH VLE	
15.00	10	*Coffee and cake - to enjoy whilst completing workshop below*			
15.10	30	*Workshop: The Improvement cycle*<br>• Teams plan out first 3 changes based upon PDSA method<br>• Use PDSA paperwork. Carol / Tim as roving faciliatators	Tim Stephens / Carol Peden	PDSA paperwork; also available on EPOCH VLE	
15.40	10	• Q&A session	Tim / Carol / Participants		
15.50	10	Evaluation and distribution of site activation packs and certificates		Site packs	

Timetable - Cluster follow-up meeting - ½ day

Time	Duration	Session	Speaker	Notes
13.00	30	Welcome and lunch	Tim	
13.30	15	Overview of trial progress and news/updates	Tim / Carol	
13.45	60	Successes and challenges so far - teams present and open discussion	Teams	Teams asked to prepare 3 slides:<br>1. Where are we now (with data)<br>2. Successes so far<br>3. Challenges so far
14.45	15	Coffee		
15.00	30	Successes and challenges continued (depending on no. of attendees)		
15.30	30	Open session - based upon learning / coaching needs of group OR action planning - teams define activity for next few months	Tim / Carol	Pre-prepared sessions about data analysis and engaging colleagues available as required<br>Action planning session available as required
16.00		Close		

EPOCH Pre - activation checklist:

- ☐ **Complete the pre-activation questionnaire and return by e-mail to qi@epochtrial.org**

- ☐ **Perform a notes review & create a 5 minute "Where we are now" site presentation to be shared at the activation meeting**
  - Obtain and review 5 sets from recent emergency laparotomies - a convenience sample is OK
  - Use the EPOCH Notes Review Tool provided to help you with this
  - Use the review to:
    - To identify care that is different from the EPOCH Care Pathway
    - To identify care that is different from your expectations of how care should be delivered
  - If possible, do this together with your other EPOCH QI leads, otherwise do individually and discuss
  - This data will form the basis of your "Emergency Laparotomy - Where we are now" presentation
  - In this presentation, include your thoughts on the major perceived barriers and opportunities for EPOCH at your hospital

- ☐ **Set up a meeting with key stakeholders, to occur shortly after activation**
  - We suggest inviting the following colleagues: (ideally at least 1 person from each staff group)
    - General and emergency surgeons – consultant grade and senior trainees
    - Anaesthetists – consultant grade and senior trainees
    - Intensivists – consultants and senior trainees
    - Emergency department representation (consultant, senior trainee or senior nurse)
    - Medical team representation
    - Clincal leads or directors
    - ODPs & Theatre nurses
    - Ward staff representation (Sister, Matron etc)
    - Service managers
    - Patient representatives
    - A member of your hospitals safety or improvement team

  It is important that the right people are in the room for this, set the meeting well in advance.

- ☐ **Start spreading the word about EPOCH and building support amongst colleagues and stakeholders**
  - EPOCH posters will be mailed out to you soon. Please put up in suitable staff-only locations
  - There are also flyers available to print, both colour and optimised for B&W.

- ☐ **Get senior leadership support for EPOCH to get the resources you need to be effective**
  - Meet with your clinical lead to discuss the possibility of allocating specific time to you for EPOCH QI activities.
  - Find an executive sponsor who will help you drive this at board level.

EPOCH All Active Sites Quality Collaborative meeting

Friday 12th of December - 9.30 until 15.45

The Education Academy, Royal London Hospital, Turner Street, London E1 1BB

This day will be a learning and review meeting for all sites activated during 2014.

**Timetable**	
**Time**	**Session**
9.30 - 10.00	**Registration**<br>*Tea/coffee and pastries*
10.00 - 10.30	**Welcome**<br>**Keynote lecture** – "How to make change happen when change is hard"<br>• ***Dr. Kevin Stewart*** - *Director of the Clinical Effectiveness and Evaluation Unit, Royal College of Physicians*
10.30 - 11.10	**Workshop** – "QI research - what does it mean to me?"
11.10- 11.35	**Break**
11.35 - 12.30	**QI Leads presentations** – "What I have learnt from EPOCH so far"
12.30 - 13.40	**Lunch**<br>*Incl. opportunities for meeting colleagues and reviewing poster presentations*
13.40 - 14.30	**Debate** – "This house believes that data is the key to QI success"<br>• ***Dr. Dave Murray*** *- Consultant Anaesthetist and Clinical Lead for NELA*<br>**vs.**<br>• ***Jonathan Bamber*** *- Research Manager, The Health Foundation*
14.30- 14.45	**Run-chart "15 minute master-class"**<br>*Effectively analyse your data for trends in just a few easy steps*
14.45 - 15.00	**Coffee and cake**
15.00 - 15.20	**QI Surgery** -<br>*Bring your questions and problems to the QI Drs.!*
15.20 - 15.30	**Summing up** - "What we have learnt from our first year of running EPOCH"<br>• ***Dr Carol Peden*** **-** *Consultant in Anaesthesia and Critical Care Medicine and EPOCH QI Lead*
15.30 - 15.45	**Poster prize, evaluation and close**

EPOCH All Active Clusters Quality Collaborative meeting

11th June 2015 - St. Bartholomews Hospital

19th June 2015 - Bradford Teaching Hospital

09.30 - 16.00

Session	Time	Presenter ([1]London [2]Bradford)
**Registration and coffee**	9.30 - 10.00	
**Welcome**	10.00 - 10.20	Prof. Carol Peden / Tim Stephens
**NELA update**	10.20 - 11.00	[1] Dr. Matt Oliver (NELA)<br><br>[2] Prof. Carol Peden
**Improving Sepsis Care**	11.00 - 11.45	[1]Sarah Stanley (RFH, London)<br><br>[2]Philip Stanley (BTH, Bradford)
**Group work**	11.45 - 12.10	All
**Lunch**	12.10 - 13.10	
**World Café session**<br><br>**(Sharing success stories)**	13.10 - 14.40	[1,2] Alex Venditelli (The Salon:Collective),<br>Tim and Carol,<br>All
**Coffee**	14.30 - 14.50	
**Interpreting your NELA Data**	14.50 - 15.30	[1]Tim Stephens<br><br>[2] Dr. Dave Saunders (RVI, Newcastle)
**Open session**	15.30 - 15.50	
**Q & A**	15.50 - 16.15	Carol Peden / Tim Stephens
**Final words, Evaluation and close**	16.30	Carol Peden / Tim Stephens

EPOCH Notes Review tool

Pre-op decision making	Y/N/NA/NK*
Consultant review documented	
Timely CT scan (within 2 hours of decision to perform test)	
**Documented mortality risk estimate pre-op using P-possum**	
Patients & relatives provided with written information	
**Pre-op interventions**	**Y/N/NA/NK**
**Sepsis Six** 1. O2 /2. Fluid bolus /3. Blood cultues /4. Abx / 5. Lacate measured /6. Fluid balance)	
Analgesia within 1 hour of first assessment	
Screening for and correction of coagulopathy	
Normothermia maintained	
Active glucose monitoring & management	
**Surgery within 6 hours of decision to operate**	

Intra-op basic care	Y/N/NA/NK
WHO checklist performed	
Normothermia maintained	
Appropriate antibiotic therapy given	
Active glucose monitoring & management	
Appropriate post-operative medications prescribed by peri-op team?<br>• Analgesia<br>• Nausea & vomiting prophylaxis<br>• VTE prophylaxis	
**Intra-op advanced care**	**Y/N/NA/NK**
Consultant delivered surgery	
Consultant delivered anaesthesia	
**Fluid therapy guided by cardiac output monitoring**	
Low tidal volume protective ventilation used	
Arterial blood gases and serum lactate measured	
**Admitted to Critical Care ≤6hrs post-op**	
**End of surgery risk evaluation**	**Y/N/NA/NK**
Serum lactate measured	
Full reversal of NMB confirmed	
Core temperature documented	
Mortality risk estimate re-evaluated	
Fluid management plan documented	

epoch better care for surgical patients

Post-op care checklist	Y/N/NA/NK
Chest physiotherapy review on day one after surgery	
Nutrition: early review with consideration of benefits of enteral feeding	
Analgesia: early review by acute pain team	
Continued antibiotic therapy (where indicated)	
Microbiology review	
Prophylaxis for post-operative nausea & vomiting prescibed	
VTE prophylaxis prescribed	
Normothermia maintained	
Active glucose management	
Daily haematology & biochemistry until mortality risk is low (senior opinion)	
Critical Care Outreach review on ward	
Early Warning Scores used	

***Y=Yes N=No NA = not applicable / appropriate NK = not known**

Map

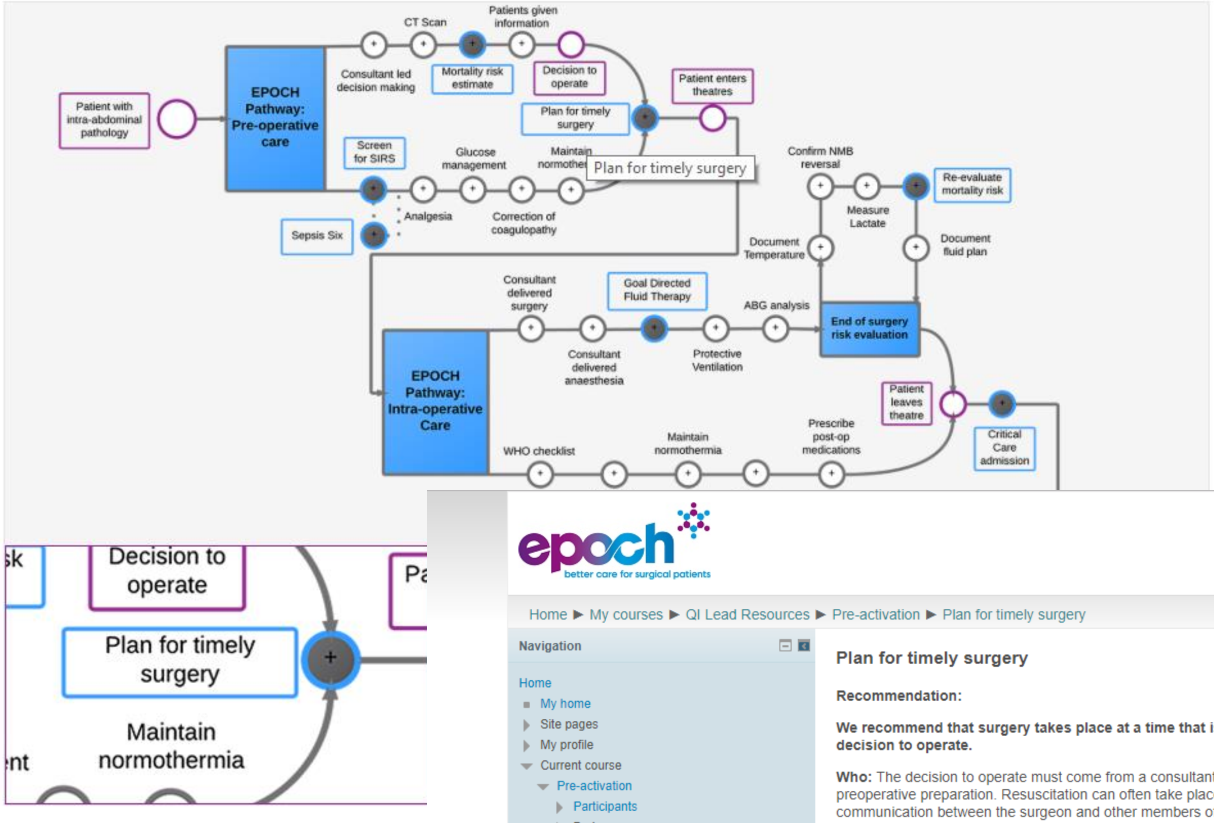

Example of Interactive EPOCH Routemap on the Virtual Learning Environment: each node on the routemap would link to a page with an explanation of and evidence for the intervention.

Here "Plan for timely surgery" is selected.

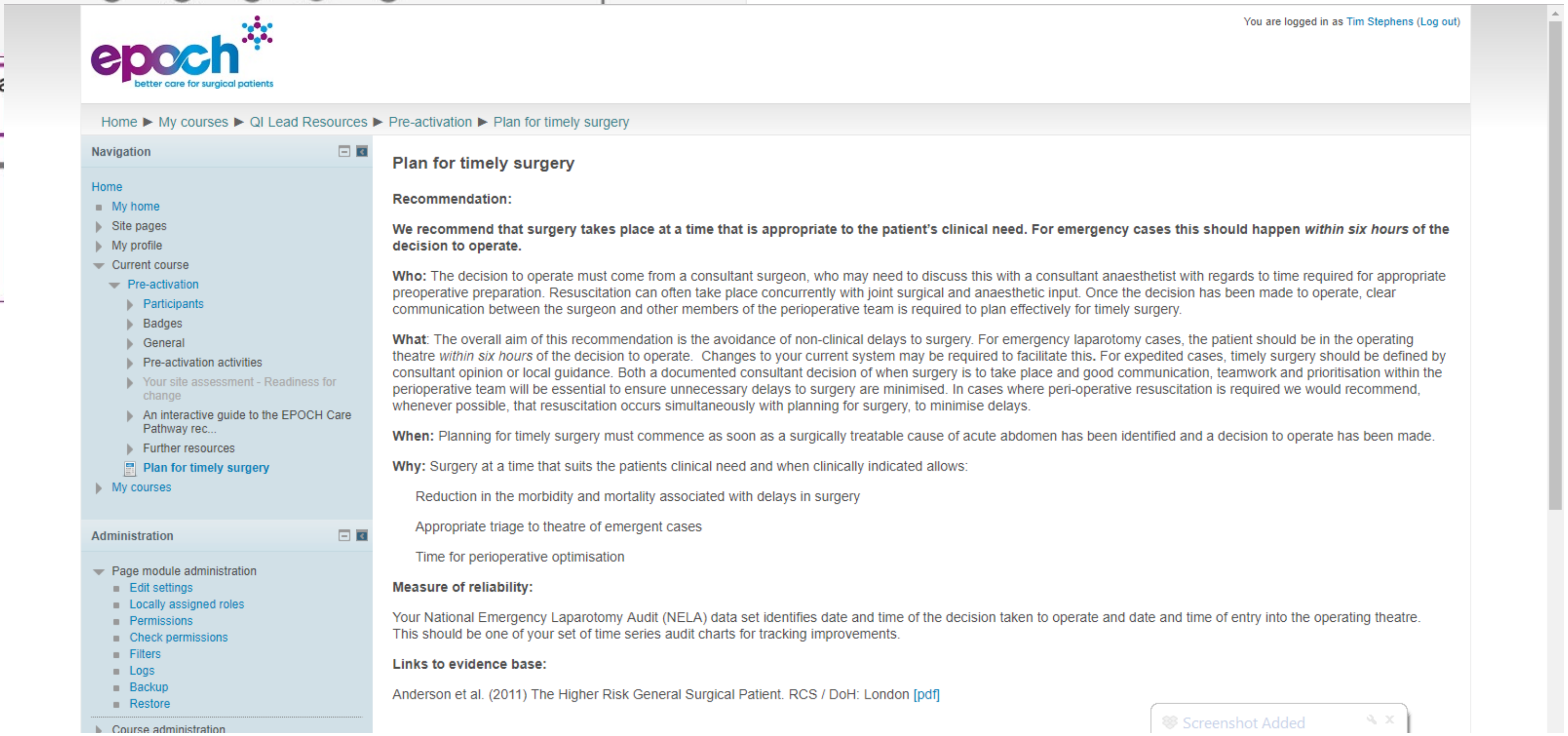

epoch
better care for surgical patients

EPOCH Trial Exit Questionnaire

1. Instructions and purpose

Thank you for taking the time to complete this exit questionnaire. The data you submit will allow us to place into context the results from the main study. As such, your answers are vital to the interpretation of our data.
Some specific points to note:

1. Completion of this questionnaire should be led by the PI or the QI Lead most involved in the running of EPOCH at your site. However, all those involved in EPOCH QI activities should ideally be given the opportunity to contribute to responses.
You may find completing the questionnaire as a team offers the chance to reflect on progress to date.

2. The first section is about the clinical interventions within the EPOCH Pathway. (*This section will take the longest to complete.)* The following sections focus on the QI activities undertaken as part of the study. There will be then be an opportunity to give the EPOCH Team some feedback.
The final section of the form is about the person completing this form and any others who have contributed.

3. This questionnaire should between 15-30 minutes to complete, depending on how many questions you answer.
We appreciate this is a signifcant time committment. Questions with a red * are required responses (you cannot progress until answered) with all others being optional. Progress can be saved at any time, if you need to stop and continue at a later date.

Although the questionnaire is not anonymous, no individual hospital level data will be presented or published.

Thank you again for your valuable input.

Carol, Rupert, Tim AND The EPOCH Trial team

1. Hospital (study site) name *

2. The EPOCH Clinical intervention

During the trial, hospitals were given some flexiblity as to what clinical interventions & care proceesses to focus on.

In this first section please indicate which clinical interventions and processes from the EPOCH pathway were included in your hospitals improvement activities, once you started the EPOCH study period.

You will then be asked to describe which interventions you found easy to implement and which were more challenging.

2. Using the list below, please indicate which of the <u>pre-operative</u> EPOCH Care Pathway interventions and processes you attempted to improve or implement during EPOCH *

	Attempted to improve / implement during EPOCH	Did not attempt to improve / implement during EPOCH	Did not attempt to improve / implement: already reliably delivered prior to EPOCH
PRE- OP Consultant-led decision making	☐	☐	☐
PRE-OP CT Scan within 2 hours of decision to image	☐	☐	☐
PRE-OP Documented mortality risk estimate using formal risk estimate tool (e.g. P-Possum)	☐	☐	☐
PRE-OP Patient and relatives given written and oral information about treatment	☐	☐	☐
PRE-OP Timely surgery	☐	☐	☐
PRE-OP Screening for Sepsis and use of Sepsis 6 as appropriate	☐	☐	☐

	Attempted to improve / implement during EPOCH	Did not attempt to improve / implement during EPOCH	Did not attempt to improve / implement: already reliably delivered prior to EPOCH
PRE-OP Analgesia within 1 hour of first medical assessment	☐	☐	☐
PRE-OP Screening for coagulopathy and correction as appropriate	☐	☐	☐
PRE-OP Maintenance of normothermia	☐	☐	☐
PRE-OP Active glucose control	☐	☐	☐

3. The EPOCH Clinical intervention

3. Using the list below, please indicate which of the <u>intra-operative</u> EPOCH Care Pathway interventions and processes you attempted to improve or implement during EPOCH *

	Attempted to improve / implement during EPOCH	Did not attempt to improve / implement during EPOCH	Did not attempt to improve / implement: already reliably delivered prior to EPOCH
INTRA-OP Consultant delivered surgery	☐	☐	☐
INTRA-OP Consultant delivered anaesthesia	☐	☐	☐
INTRA-OP Fluid guided by CO monitoring	☐	☐	☐
INTRA-OP Low tidal volume protective ventilation	☐	☐	☐
INTRA-OP Serum lactate analysis	☐	☐	☐
INTRA-OP Plan for Critical Care admission	☐	☐	☐
INTRA-OP WHO checklist performed	☐	☐	☐
INTRA-OP Screening for Sepsis and use of appropriate antibiotic therapy	☐	☐	☐
INTRA-OP Maintenance of normothermia	☐	☐	☐
INTRA-OP Active glucose management	☐	☐	☐
INTRA-OP Post-operative analgesia and VTE / N&V prophylaxis prescribed	☐	☐	☐

4. The EPOCH Clinical intervention

4. Using the list below, please indicate which of the 'End of Surgery ' EPOCH Care Pathway interventions and processes you attempted to improve or implement during EPOCH *

	Attempted to improve / implement during EPOCH	Did not attempt to improve / implement during EPOCH	Did not attempt to improve / implement: already reliably delivered prior to EPOCH
END OF SURGERY Document core temperature	☐	☐	☐
END OF SURGERY Confirm neuromuscular blockade reversal using stimulation device	☐	☐	☐
END OF SURGERY Re-check serum lactate	☐	☐	☐
END OF SURGERY Re-calculate mortality risk estimate using formal tool (e.g. P-Possum)	☐	☐	☐
END OF SURGERY Document fluids given and fluid plan	☐	☐	☐

5. The EPOCH Clinical intervention

5. Using the list below, please indicate which of the post-operative EPOCH Care Pathway interventions and processes you attempted to improve or implement during EPOCH *

	Attempted to improve / implement during EPOCH	Did not attempt to improve / implement during EPOCH	Did not attempt to improve / implement: already reliably delivered prior to EPOCH
POST-OP Early pain team review	☐	☐	☐
POST-OP Continued antibiotic therapy with microbiology input	☐	☐	☐
POST-OP Early nutrition review (surgical / dietician led)	☐	☐	☐
POST-OP Physiotherapy on Day 1 after surgery	☐	☐	☐
POST-OP Maintenance of normothermia	☐	☐	☐
POST-OP Active glucose management	☐	☐	☐
POST-OP Daily bloods taken until considered low risk	☐	☐	☐
POST-OP Nausea and vomiting prophylaxis given	☐	☐	☐
POST-OP VTE prophylaxis given	☐	☐	☐
POST-OP Critical Care Outreach Team review on ward	☐	☐	☐

Please use this space to add any further information you feel is relevant regarding the clinical interventions

6. The EPOCH Clinical intervention

6. Please indicate statement most closely fits your hospitals improvement or implementation activity during EPOCH *

- [ ] We introduced a single pathway of care (across Pre, Intra and Post operative phases)
- [ ] We introduced separate pathways or care bundles for 2 or more phases of the patient admission (e.g. a pre-op pathway plus an intra op care bundle)
- [ ] We introduced separate pathways or bundles for one phase of the patient admission (e.g. pre-op or post op only)
- [ ] We focused on introducing individual / separate interventions
- [ ] Other (please specify):

7. The EPOCH Clinical intervention

7. Please tell us which interventions were easiest to implement / improve and why this was

8. Please tell us which interventions were most challenging to implement / improve and why this was

Well done! That is the hardest and longest part completed.
Now onto some questions about your QI activities.
The majority of these are simple Yes / No or multi-choice questions.

8. Quality Improvement (QI) activities

This second section will cover what QI activities and strategies were used by you and your colleagues when improving care for Emergency Laparotomy patients

9. At your site, was a formal team created to work on QI activities related to EPOCH?

Definition of QI Team:
A group of individuals that work together on the QI project. The team is defined by their shared goals and mutual accountability for the QI project outcome. QI team members are typically responsible for planning and conducting tests of change and/or data collection and management. Members of the QI team may be anyone within the healthcare team, such as doctors, nurses, AHPs, pharmacists, managers, administrative staff. *

- [ ] Yes
- [ ] No
- [ ] Other (please specify):

9. Quality Improvement (QI) activities (continued)

10. Please indicate the approximate size of your QI Team, including yourself *

- [ ] 1
- [ ] 2
- [ ] 3
- [ ] 4
- [ ] 5
- [ ] 6
- [ ] 7
- [ ] 8
- [ ] 9
- [ ] 10
- [ ] >10

11. Which professions and disciplines were involved in your QI Team, including yourself? *

- [ ] Surgeons
- [ ] Anaesthetists
- [ ] Intensivists
- [ ] Radiologists

- [ ] Acute Medicine
- [ ] Emergency Medicine
- [ ] Healthcare of the Elderly physicans
- [ ] Surgeons in training
- [ ] Anaesthetists in training
- [ ] Other doctor in training
- [ ] Nursing - theatres
- [ ] Nursing - ward / critical care
- [ ] Nursing - research (any speciality)
- [ ] Operating Department Practitioners
- [ ] Other Allied Health Professionals
- [ ] Service / departmental managers
- [ ] Senior / executive management
- [ ] Audit / data staff
- [ ] Other (please specify):

10. Quality Improvement (QI) activities (continued)

12. Please indicate which of the methods below best describes your NELA data collection process
N.B. If your process has changed significantly over time, pleas indicate which method is in use now and use the comment box to briefly describe this change. *

- [ ] Mostly prospective - the majority of data are collected concurrently and in real time during the peri-operative period by members of the team delivering patient care
- [ ] Mostly retrospective - the majority of data are collected after the peri-operative period by members of the team responsible for that patient care episode
- [ ] Most retrospective - the majority of data are collected after the peri-operative period by other staff not directly involved in that patient care episode
- [ ] Other (please specify):

Comments:

13. Who enters most of the data into the NELA online portal? (you may choose up to 3 options) *

- [ ] EPOCH QI Leads
- [ ] NELA Leads
- [ ] Other clinicians (Consultant grade)
- [ ] Other clinicians (in training)
- [ ] Nursing staff - clinical

☐ Nursing staff - research

☐ Allied Health Professionals

☐ Audit / data staff

☐ Other (please specify):

Comments:

14. Were data collected on care processes NOT captured by NELA?
e.g. Sepsis screening *

☐ Yes

☐ No

If YES, please describe briefly:

15. Prior to starting EPOCH did you or your colleagues download and analyse your local NELA data *

☐ Yes

☐ No

☐ Other (please specify):

If YES, please describe briefly:

16. After starting EPOCH did you or your colleagues download and analyse your local NELA data? *

☐ Yes

☐ No

11. Quality Improvement (QI) activities (continued)

17. What methods did you or your colleagues use to analyse and display your local NELA data? *

- [ ] Run charts
- [ ] Statical Process Control (SPC) charts
- [ ] Bar charts
- [ ] Pie charts
- [ ] Summary statistics
- [ ] Red Amber Green ('RAG') status charts
- [ ] Other (please specify):

12. Quality Improvement (QI) activities (continued)

18. Please indicate approximately how frequently you or your colleagues analysed your local NELA data? *

- ☐ Weekly
- ☐ Fortnightly
- ☐ Monthly
- ☐ Bi-monthly
- ☐ Every 3-4 months
- ☐ Every 6 months
- ☐ Only once - did not update
- ☐ Other (please specify):

13. Quality Improvement (QI) activities (continued)

19. Please use the scale below to rate your agreement with the following statement: "I / we found run-charts helpful when analysing and interpreting our NELA data" *

- [ ] Strongly agree
- [ ] Agree
- [ ] Neutral
- [ ] Disagree
- [ ] Strongly disagree

It would be helpful if you could provide a brief reason for your rating

14. Quality Improvement (QI) activities (continued)

20. Did you feedback your NELA data and analysis to colleagues during EPOCH? *

- [ ] Yes
- [ ] No

Comments:

15. Quality Improvement (QI) activities (continued)

21. Please take a moment to describe how you fedback data to colleagues.
Please include:
1. Who you fedback data to;
2. How frequently you did this;
3. What methods you used (e.g. email, EPOCH meetings, departmental events/meetings, posters)
4. Any other activities relevant to feedback of NELA data to colleagues *

22. Please use the scale below to rate your agreement with the following statement:
"I / we found run-charts helpful when feeding back NELA data to other colleagues" *

- [ ] Strongly agree
- [ ] Agree
- [ ] Neutral
- [ ] Disagree
- [ ] Strongly disagree
- [ ] Did not use runcharts

It would be helpful if you could provide a brief reason for your rating

23. Please use the scale below to rate your agreement with the following statement: "From my / our experience during EPOCH, feeding back data to colleagues can be an effective strategy to motivate those colleagues to improve care " *

- [ ] Strongly Agree
- [ ] Agree
- [ ] Neutral
- [ ] Disagree
- [ ] Strongly Disagree

Please provide a brief reason for your rating

16. Quality Improvement (QI) activities (continued)

24. Please summarise any board level support you received during the study period

17. Quality Improvement (QI) activities (continued)

25. Did you hold a stakeholder meeting as one of your QI activities?
e.g. a meeting for all professionals involved in the care of EmLap patients *

- [ ] Yes
- [ ] No

If YES, please describe briefly:

26. Did you or your colleagues use the "Plan Do Study Act" (PDSA) cycle approach during your QI activities? *

- [ ] Yes, often
- [ ] Yes, occasionally
- [ ] No

18. Quality Improvement (QI) activities (continued)

27. Please use the scale below to rate your agreement with the following statement:
" I / we found the PDSA cycle to be a helpful approach to implementation / improvement" *

- [ ] Strongly Agree
- [ ] Agree
- [ ] Neutral
- [ ] Disagree
- [ ] Strongly Disagree

Comments:

19. Your experience of improving care

Nearly done!
In this section, please take a moment to tell us your thoughts on what has worked and not worked for you during EPOCH

28. Reflecting on your experience with EPOCH, please tell us what are the 2 things that you would definitely continue doing if you were to do EPOCH again. *

29. Reflecting on your experience with EPOCH, please tell us 2 things you would do differently if you were to do EPOCH again. *

30. The EPOCH Theory of Change was based upon several key interventions.
From your experience with EPOCH please rank these in order of importance.
N.B. The 4 choices will move with your ranking decisions. *

Using data to drive improvement

Creating the motivation and will to change amongst stakeholders

Fostering inter-professional collaboration and team working

Using QI methods (such as the PDSA cycle) to improve care

Comments:

31. You can use this space to tell us more about the barriers and enablers of improvement you have experienced during EPOCH

20. Feedback to the EPOCH Trial team

32. Please rate the support available to you during the EPOCH Trial from the trial team *

- ☐ Very good
- ☐ Good
- ☐ Acceptable
- ☐ Poor
- ☐ Very poor

33. Please tell us what we did that you found helpful

34. Please tell us what we could have done better

35. Please enter your email address here *

36. If others contributed to these responses please list their name and profession or job title here.

37. Would you be willing to be contacted by the EPOCH Trial team to discuss your answers in more detail?

- [ ] Yes
- [ ] No
